# Valorisation of lignocellulose and low concentration CO_2_ using a fractionation–photocatalysis–electrolysis process†

**DOI:** 10.1039/d3gc03258b

**Published:** 2023-11-13

**Authors:** Santiago Rodríguez-Jiménez, Erwin Lam, Subhajit Bhattacharjee, Erwin Reisner

**Affiliations:** a Yusuf Hamied Department of Chemistry, University of Cambridge Lensfield Road CB2 1EW Cambridge UK reisner@ch.cam.ac.uk

## Abstract

The simultaneous upcycling of all components in lignocellulosic biomass and the greenhouse gas CO_2_ presents an attractive opportunity to synthesise sustainable and valuable chemicals. However, this approach is challenging to realise due to the difficulty of implementing a solution process to convert a robust and complex solid (lignocellulose) together with a barely soluble and stable gas (CO_2_). Herein, we present the complete oxidative valorisation of lignocellulose coupled to the reduction of low concentration CO_2_ through a three-stage fractionation–photocatalysis–electrolysis process. Lignocellulose from white birch wood was first pre-treated using an acidic solution to generate predominantly cellulosic- and lignin-based fractions. The solid cellulosic-based fraction was solubilised using cellulase (a cellulose depolymerising enzyme), followed by photocatalytic oxidation to formate with concomitant reduction of CO_2_ to syngas (a gas mixture of CO and H_2_) using a phosphonate-containing cobalt(ii) bis(terpyridine) catalyst immobilised onto TiO_2_ nanoparticles. Photocatalysis generated 27.9 ± 2.0 μmol_CO_ g_TiO_2__^−1^ (TON_CO_ = 2.8 ± 0.2; 16% CO selectivity) and 147.7 ± 12.0 μmol_formate_ g_TiO_2__^−1^ after 24 h solar light irradiation under 20 vol% CO_2_ in N_2_. The soluble lignin-based fraction was oxidised in an electrolyser to the value-added chemicals vanillin (0.62 g kg_lignin_^−1^) and syringaldehyde (1.65 g kg_lignin_^−1^) at the anode, while diluted CO_2_ (20 vol%) was converted to CO (20.5 ± 0.2 μmol_CO_ cm^−2^ in 4 h) at a Co(ii) porphyrin catalyst modified cathode (TON_CO_ = 707 ± 7; 78% CO selectivity) at an applied voltage of −3 V. We thus demonstrate the complete valorisation of solid and a gaseous waste stream in a liquid phase process by combining fractioning, photo- and electrocatalysis using molecular hybrid nanomaterials assembled from earth abundant elements.

## Abstract 2

This work demonstrates the simultaneous upcycling of all components in lignocellulosic biomass together with the greenhouse gas CO_2_ as an attractive opportunity to synthesise sustainable and valuable chemicals.

## Graphical abstract

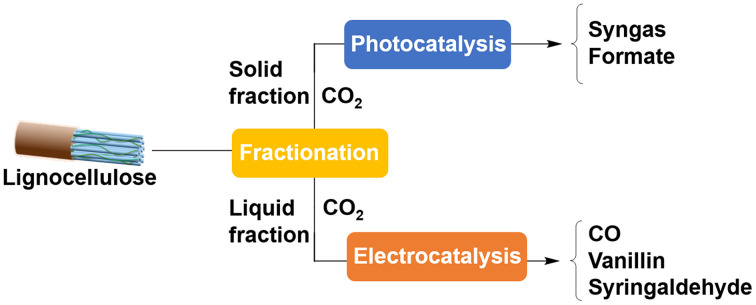

## Introduction

The renewable generation of valuable chemicals and fuels is a critical step towards a sustainable chemical industry.^1,2^ The valorisation of abundant waste resources, such as lignocellulosic biomass and the greenhouse gas CO_2_ offers great potential to achieve such an ambitious goal at the scale required to defossilise our industry. By harnessing the power of renewable energy sources, such as solar and wind, the conversion of non-edible biomass and CO_2_*via* photo- or electrochemical approaches presents an opportunity to produce sustainable fuels and chemicals.^3^

Lignocellulosic biomass such as wood, is abundant and cheap and consists predominantly of three polymeric components: cellulose, hemicellulose and lignin.^4,5^ Cellulose and hemicellulose consist mainly of polysaccharide made from glucose, xylose, mannose and arabinose. Lignin is made of different polymerised aromatic units, and its utilisation remains challenging due to the robustness of its polyaromatic structure, which requires harsh conditions to break down the polymer (*e.g.*, strong acids such as H_2_SO_4_).^6,7^ Unlike cellulose, lignin-to-chemical conversion technologies remain scarce.^4,5,7,8^ An important linkage in lignin, often found between its aromatic polymeric backbone, is the β-O-4 bond between two phenyl rings, which serves as an ideal target to depolymerise lignin into smaller aromatic fragments.^9–11^ The selective depolymerisation of all lignocellulose components and their subsequent chemical transformation would enable large-scale access to aliphatic and aromatic renewable feedstock chemicals.

Apart from ubiquitous biomass sources, the greenhouse gas CO_2_ can be used as an abundant carbon source to produce energy-rich chemicals such as CO, formate, hydrocarbons or alcohols.^12^ However, photo- and electroreduction of CO_2_ are predominantly performed in the presence of pure CO_2_, where concentrated CO_2_ streams have to be generated involving additional energy input.^13–15^ To alleviate the energy demand of the process, it is desirable to perform catalytic reactions at lower CO_2_ concentrations (*e.g.*, ≤20% CO_2_).^16–20^ The challenge of using low concentration CO_2_ streams lies in maintaining high product selectivity and catalytic activity compared to reactions employing pure CO_2_ streams.^17^ Molecular CO_2_ reduction catalysts display an increased product selectivity compared to most heterogeneous electro- or photocatalysts.^16,21,22^

An attractive approach to utilise biomass and CO_2_ together is their simultaneous conversion in photo- or electrocatalytic processes.^23–25^ This strategy opens the possibility to couple biomass oxidation with CO_2_ reduction in a single process driven by sunlight or renewable electricity. The coupling of productive half-reactions thereby allows the conversion of a solid and a gaseous waste stream into valuable products such as CO, syngas, formate and aromatic chemicals, which can be more attractive than conventional systems performing overall water splitting to generate H_2_ and O_2_ from water. Additionally, the oxidation of biomass-derived substrates is thermodynamically less demanding than water oxidation, thereby facilitating the catalytic conversions, as well as the generation of value-added products.^26–28^ This combined approach also allows for the isolation of products in different phases and compartments, which can help with product separation. Conventional approaches such as water splitting generate explosive H_2_ and O_2_ mixtures in the reactor headspace.

The valorisation of cellulose and CO_2_ streams has recently been reported using a TiO_2_ nanoparticle with an immobilised CO_2_ reducing cobalt(ii) bis(terpyridine) catalyst containing phosphonate anchors (CotpyP) (TiO_2_∣CotpyP). Photoexcitation of this hybrid TiO_2_∣CotpyP photocatalyst reduced aqueous CO_2_ to syngas, while cellulose-derived glucose was simultaneously oxidised to formate and arabinose. TiO_2_∣CotpyP could operate for 24 h and be recycled up to three times.^29^ However, only concentrated CO_2_ and pure cellulose have been used, and no strategy for lignin separation and utilisation were reported in this previous study.

**Fig. 1:**
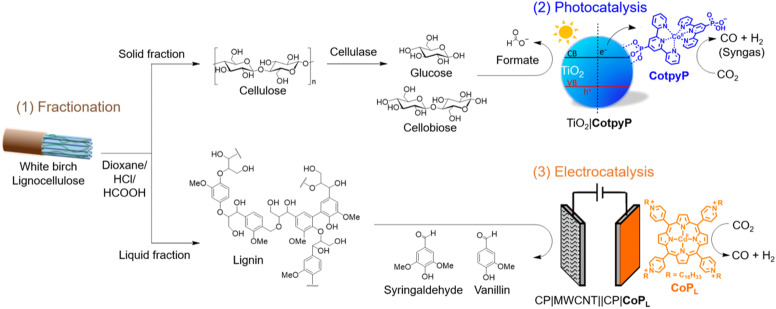
Schematic overview of a three-stage process for the complete valorisation of lignocellulose and diluted CO_2_. (1) Fractionation of lignocellulose from white birch into a predominant cellulose and lignin fraction (top and bottom, respectively), with the cellulose-based solid fraction being further incubated in cellulase to generate a soluble sugar (glucose and cellobiose) solution. Structures of xylose, mannose and arabinose are omitted for clarity. (2) Photocatalytic oxidation of cellulose-derived glucose and cellobiose to formate coupled with reduction of diluted CO_2_ to syngas (CO and H_2_) using TiO_2_∣CoptyP photocatalyst. (3) Electrolytic oxidation of lignin to aromatic aldehydes syringaldehyde and vanillin coupled with reduction of diluted CO_2_ to CO and H_2_ using CP∣MWCNT∣∣CP∣CoP_L_.

Herein, we report the valorisation of all components in lignocellulose coupled to low concentration CO_2_ reduction, which has been achieved by employing molecular CO_2_ reduction hybrid nanomaterials (Fig. 1). First, lignocellulose is pre-treated and fractioned into predominantly cellulosic- and lignin-based components using acid hydrolysis. Second, the fractionated cellulosic solution was converted with low concentration CO_2_ using the TiO_2_∣CotpyP photocatalyst to HCOO^−^ and syngas, respectively. Finally, an electrolysis process concomitantly converted the fractionated lignin solution on a carbon-based anode to vanillin and syringaldehyde, which find application in the food, pharma and cosmetics industries.^10,30^ Diluted CO_2_ is reduced to CO (with a single pass conversion yield close to 5% at both 10 vol% and 20 vol% CO_2_) with a molecular cobalt(ii) porphyrin (CoP_L_) catalyst immobilised on a multiwall carbon nanotube (MWCNT) cathode. Thus, we demonstrate the complete valorisation of lignocellulose and low concentration CO_2_, which has been enabled by a precious-metal free fractionation–photocatalysis–electrolysis process.

## Results and discussion

### Fractionation of lignocellulose

The composition of dried and extracted white birch wood used in this study was determined by quantifying its sugar and lignin composition following standard characterisation procedures using H_2_SO_4_ hydrolysis:^6^ glucose (34.7 ± 1.6 wt%), xylose (20.3 ± 1.0 wt%), mannose (1.8 ± 0.1 wt%), arabinose (0.9 ± 0.1 wt%) and lignin (17.6 ± 1.4 wt%). Further details are provided in the Experimental section, Fig. 1, Fig. S1 and Tables S1 and S2.†

To effectively utilise the different components of lignocellulose, their individual components are first separated by acid hydrolysis. Lignocellulose was pre-treated in a dioxane/HCl/HCOOH mixture at 80 °C for 3 h (500 mg in 5.9 mL) to obtain a liquid (or liquor) and a solid fraction.^7^ The dioxane/HCl/HCOOH mixture solubilises lignin and the liquid fraction predominantly consisted of lignin (41.2 ± 2.1 wt%) along with xylose (6.9 ± 1.3 wt%), glucose (2.4 ± 0.6 wt%), mannose (1.3 ± 0.3 wt%) and arabinose (2.5 ± 0.7 wt%). The second most abundant component xylose (derived from hemicellulose) is partially converted to furfural under these fractioning conditions (see Experimental section, and Fig. 1, Fig. S1 and Tables S1–S3† for further details).^26^ The isolated solid fraction consisted mainly of cellulosic components such as glucose (62.9 ± 1.9 wt%) and xylose (6.7 ± 1.6 wt%) with some lignin (5.4 ± 1.2 wt%).

### Photocatalytic valorisation of cellulose and CO_2_

The solid fraction obtained from lignocellulose fractionation contained mainly cellulose and was used as a feedstock in photocatalysis following depolymerisation *via* cellulase pre-treatment.^29^ The depolymerised glucose and cellobiose are suitable electron donors in semiconductor suspension systems,^31^ and their valorisation has been previously reported.^32,33^ Specifically, the solid fraction was enzyme pre-treated with cellulase (0.05 mg_cellulase_ mg_solid_^−1^) to generate soluble sugars, predominantly glucose (39.3 ± 4.7 mM) and cellobiose (8.7 ± 2.1 mM) after 24 h incubation at 37 °C in an aqueous sodium acetate buffer solution (50 mM) at pH 5.^29^ With respect to white birch, 10.2 ± 0.4 wt% and 4.2 ± 0.6 wt% were converted to glucose and cellobiose, respectively (see Tables S1 and S2†).

The lignocellulose-derived sugar solution was then utilised for CO_2_ reduction reactions using the TiO_2_∣CotpyP photocatalyst (Fig. 1).^29^ In a typical experiment, CotpyP (50 nmol) was added to a photoreactor containing a TiO_2_ suspension (5 mg, P25, particle diameter ∼20 nm) in 2 : 1 MeCN : cellulase-treated solid fraction aqueous solution (3 mL). The photoreactor was sealed with a rubber septum and purged with 100% CO_2_ or 20% CO_2_ (balanced with N_2_) at a flow rate of 15 mL min^−1^ for 15 min. The sealed and stirred photoreactor was irradiated with a solar light simulator (100 mW cm^−2^, AM 1.5G, 25 °C, 600 rpm) for 24 h. The UV in the full solar spectrum is necessary to photoexcite electrons from the valence to the conduction band of TiO_2_.^29^ The gaseous products (H_2_ and CO) in the headspace (4.74 mL) were quantified by gas chromatography (GC), and HCOO^−^ formed in the solution from glucose photooxidation was quantified by ion chromatography.

**Fig. 2:**
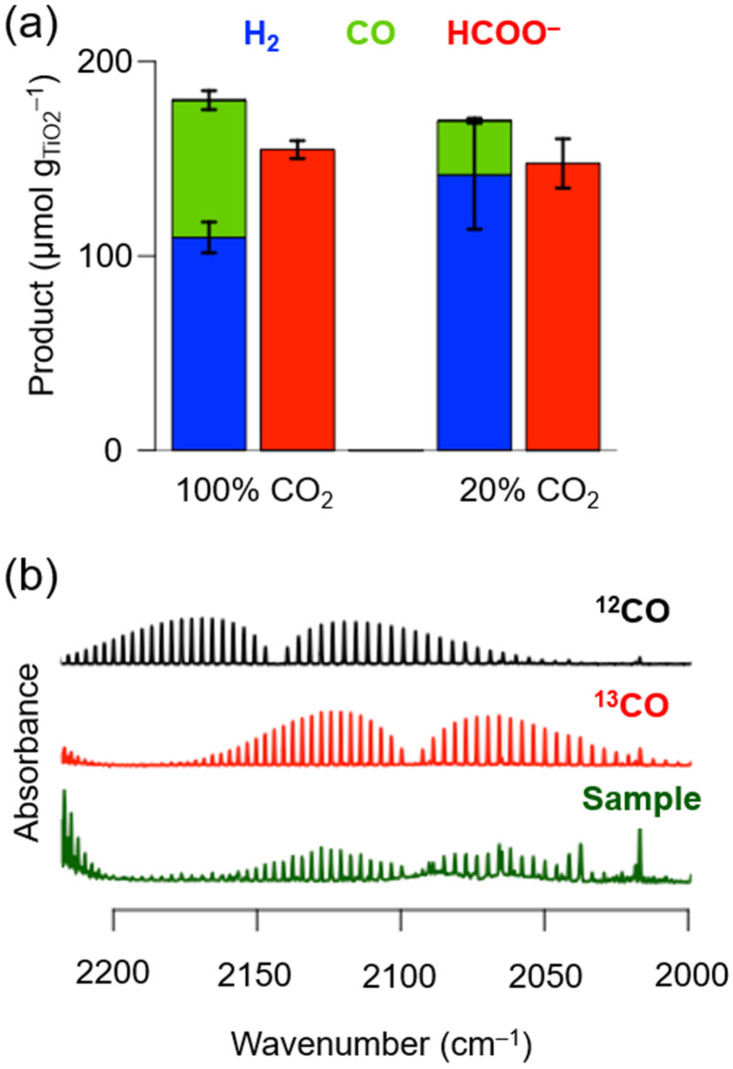
(a) H_2_, CO and formate (blue, green and red, respectively) formation after 24 h of photocatalysis with TiO_2_∣CotpyP. (b) Transmission IR spectra with ^13^CO_2_ isotopic labelling of the headspace after 24 h of photocatalysis (sample, green curve), including ^12^CO (black) and ^13^CO (red) as reference. Reaction conditions: 5 mg TiO_2_ (P25); 50 nmol CotpyP; 3 mL 2 : 1 MeCN : pre-treated reaction solution (cellulose fraction pre-treated with cellulase); 25 °C, 100 mW cm^−2^, AM 1.5G. (a) 20 vol% CO_2_ was balanced with N_2_; (b) sample (green) was purged with 100% ^13^CO_2_. Experiments in (a) were performed in triplicates.

After 24 h of photocatalysis under 100 vol% CO_2_, 69.9 ± 4.0 μmol_CO_ g_TiO_2__^−1^, 109.8 ± 8.0 μmol_H_2__ g_TiO_2__^−1^ (*i.e.*, 39% CO and 61% H_2_ selectivity for gaseous products) and 153.7 ± 4.0 μmol_formate_ g_TiO_2__^−1^ were formed. Under 20 vol% CO_2_, the formation yields were 27.9 ± 2.0 μmol_CO_ g_TiO_2__^−1^, 141.7 ± 27.9 μmol_H_2__ g_TiO_2__^−1^ (*i.e.*, 16% CO and 84% H_2_ selectivities) and 147.7 ± 12.0 μmol_formate_ g_TiO_2__^−1^ (Fig. 2a and Table S4†). The obtained CO yields correspond to a CO_2_-to-CO conversion yield of ∼0.03% and ∼0.05% at 100 vol% and 20 vol% CO_2_, respectively. The reduction of protons (from water) to H_2_ and CO_2_ to CO by CotpyP as well as the oxidation of glucose/cellobiose by TiO_2_ to formate are two-electron processes with an expected 1 : 1 stoichiometric ratio for (H_2_ + CO) : formate, which is close to the observed ratios.^29,34–36^

The carbon source of the products was confirmed by isotopic labelling experiments. Experiments with ^13^CO_2_ and cellulase enzyme pre-treated cellulose were performed to confirm that the CO originates from CO_2_ reduction (for further details see Experimental section). Analysis of the gas headspace after photocatalysis by transmission IR spectroscopy reveals that ^13^CO produced by TiO_2_∣CotpyP was only formed when ^13^CO_2_ was used as the carbon source (Fig. 2b). Furthermore, in the case of formate, based on previous work using ^13^C_6_-glucose with TiO_2_∣CotpyP, ^13^C-formate is only formed through the photooxidation of ^13^C_6_-glucose, confirming formate's carbon source.^29^ Further mechanistic insights of the photooxidation of glucose to formate can be found in ref. 36.

These results demonstrate that the cellulose solid fraction, following cellulase pre-treatment, provides a source of suitable electron donors for photocatalytic CO_2_ reduction. The solar TiO_2_∣CotpyP reforming system was able to convert CO_2_-to-CO at concentrations of 20 vol% CO_2_, with an activity drop of only a factor of two despite the five-fold drop in CO_2_ concentration with respect to 100 vol% CO_2_ (Fig. 2a). Near stoichiometric amounts of HCOO^−^ to CO/H_2_ were formed at both CO_2_ concentrations, demonstrating the effectiveness of TiO_2_∣CotpyP to photooxidise sugars to formate and concomitantly photoreduce CO_2_ and H_2_O to CO and H_2_.

### Electrolytic valorisation of lignin and CO_2_

To optimise the simultaneous electrolysis of CO_2_ and lignin, the half-reactions of electroreduction of low CO_2_ concentration and electrooxidation of lignocellulose-derived lignin were first studied individually in a three-electrode setup. This was followed by the proof-of-concept coupling of both half reactions in a two-electrode electrolyser using the optimised cathode and anode.

Low concentration CO_2_ electroreduction was performed on CoP_L_ immobilised on MWCNT as a molecular catalyst (see Fig. 1). CoP_L_ was chosen based on its known CO selectivity during electroreduction of pure CO_2_, and stability when immobilised on MWCNT *via* π–π stacking and its lipophilic alkyl chains (see ESI Note 1, and Fig. S2–S4†).^28,37^ We therefore further explored the electroreduction ability of CoP_L_ supported on MWCNT under variable CO_2_ concentrations ranging 10, 20, 50 and 100 vol% (balanced with N_2_), using an electrochemical flow setup that allowed continuous purging of the electrolyte solution with a given gas composition.^38^

Cathodes containing CoP_L_ were prepared, following a reported procedure (see Fig. S5†),^28^ by drop-casting a dimethylformamide (DMF) suspension containing 2.37 mg MWCNT mL^−1^ and 0.1 mM CoP_L_ onto carbon paper (CP) (0.1 mL_DMF_ cm^−2^, geometrical surface area = 1 cm^2^), which is denoted as CP∣CoP_L_. Electrocatalysis with CP∣CoP_L_ was performed in a two-compartment electrochemical cell with a three-electrode setup. Pt foil was used as the counter electrode (CE) and Ag/AgCl (sat. KCl) as reference electrode (RE), a Nafion membrane separating the cathode and anode chambers, with the catholyte (0.1 M NaHCO_3_ in H_2_O) under a constant gas flow (9 mL min^−1^) of CO_2_ and N_2_ regulated by mass flow controllers. The generated gaseous products during electrochemical experiments (H_2_ and CO) were measured *via* online GC (schematically represented in Fig. S6†).^38^

**Fig. 3:**
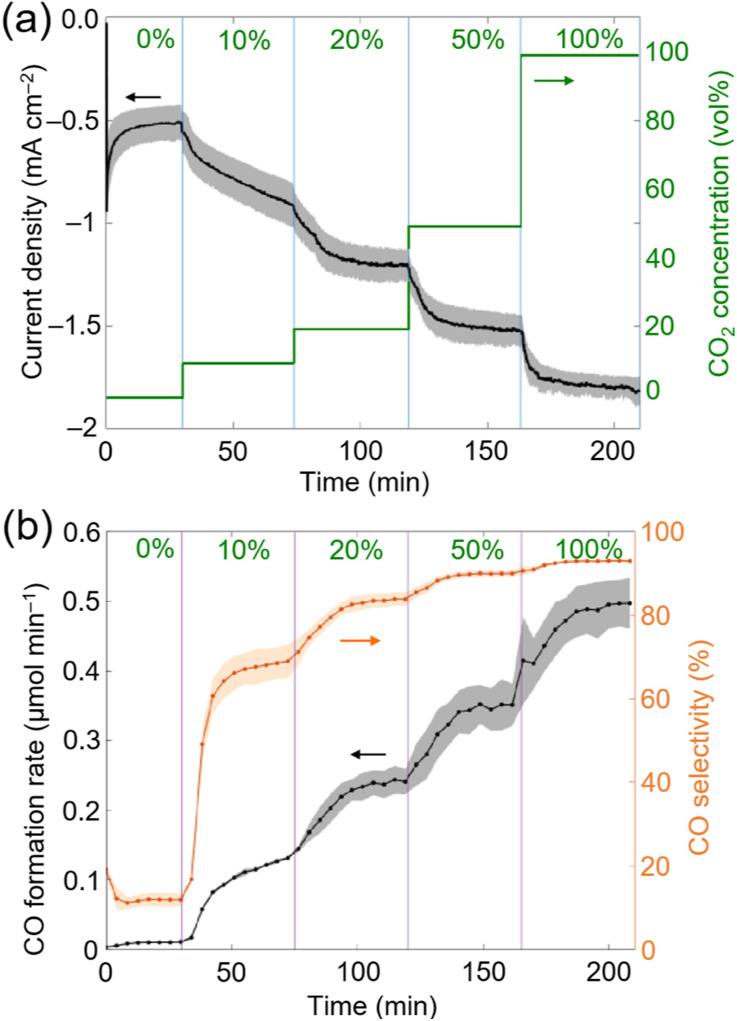
(a) Chronoamperometry (CA) experiments as a function of concentration of CO_2_ and time during CO_2_ electrochemical reduction to CO using CP∣CoP_L_ cathodes. (b) CO formation rate and selectivity during CA experiments. Experiments were performed in triplicates, and the shaded area represents the standard deviation. Reaction conditions: CA: 0.1 M NaHCO_3_ in H_2_O (pH = 6.7); *E*_app_ = −1.2 V *vs.* Ag/AgCl for 3.5 h; WE: CP∣CoP_L_, CE: Pt foil, RE: Ag/AgCl (sat. KCl). Flow of CO_2_ : N_2_ 9 mL min^−1^. CO_2_ concentration was varied stepwise between 0 vol%, 10 vol%, 20 vol%, 50 vol% and 100 vol% and balanced with N_2_.

During chronoamperometry (CA) experiments, under an applied potential of −1.2 V *vs.* Ag/AgCl (sat. KCl) the catholyte chamber was continuously purged with pure N_2_ for 30 min, after which the CO_2_ concentration was gradually increased to 10, 20, 50 and 100 vol% every 45 min (Fig. 3a). Under pure N_2_, the current density was the lowest at approximately −0.5 mA cm^−2^ and around ∼0.08 μmol_H_2__ min^−1^ evolved as the main gaseous product, with a minor CO background (<0.01 μmol_CO_ min^−1^) likely caused by the chemical equilibrium between carbonic acid (H_2_CO_3_) and CO_2_. Upon increasing the CO_2_ concentration (in vol%), the current density gradually increased, from approximately −0.8 mA cm^−2^ (10 vol%), to −1.2 mA cm^−2^ (20 vol%), to −1.5 mA cm^−2^ (50 vol%) and to −1.8 mA cm^−2^ (100 vol%). The H_2_ formation rate remained constant at ∼0.06 μmol_H_2__ min^−1^ at all CO_2_ concentration steps, whereas the CO formation rate and CO selectivity increased from ∼0.12 μmol_CO_ min^−1^ and ∼65% (10 vol%), to ∼0.24 μmol_CO_ min^−1^ and ∼80% (20 vol%), to ∼0.35 μmol_CO_ min^−1^ and ∼90% (50 vol%) and to ∼0.50 μmol_CO_ min^−1^ and ∼93% (100 vol%) (Fig. 3b). The low H_2_ formation rate across all studied CO_2_ concentrations (∼0.06 μmol_H_2__ min^−1^) may be explained by the high affinity of CoP_L_ to CO_2_.^28^ The carbon source of CO was previously confirmed for CoP_L_ through ^13^CO_2_ isotopic labelling experiments.^28,37^

Two main observations can be made by screening different CO_2_ gas flow concentrations: (1) CoP_L_ exhibits a high product specificity with around ∼80% CO selectivity even under 20 vol% of substrate CO_2_. (2) The CO formation rate at 20 vol% CO_2_ (∼0.24 μmol_CO_ min^−1^; CO turnover frequency (TOF_CO_) = 8.3 min^−1^; single-pass CO_2_ conversion yield^39^ = 4.7%) corresponds to roughly half the activity with respect to 100 vol% CO_2_ (∼0.50 μmol_CO_ min^−1^; TOF_CO_ = 17.2 min^−1^; single-pass CO_2_ conversion yield = 2.0%), thus indicating that decreasing five-fold the CO_2_ concentration only reduces two-fold the CO formation rate (Fig. 3b).

When comparing CP∣CoP_L_ with TiO_2_∣CotpyP, both molecular hybrid systems exhibit similar CO formation rate trends, although CP∣CoP_L_ maintains higher CO selectivity across all CO_2_ concentrations (see ESI Note 1 and Tables S4 and S5†). The origin of the observed trends for TiO_2_∣CotpyP and CP∣CoP_L_ at different CO_2_ concentrations remains unclear but may be attributed to their molecular structure, which provides intrinsic affinity towards CO_2_. In comparison with state-of-the-art molecular systems, such as the rhenium bipyridine electrocatalyst [Re(4,4-dimethyl-2,2-dipyridyl)(CO)_3_(triethanolamine)], which operates in a DMF/triethanolamine solvent mixture at variable CO_2_ concentrations (1, 10 and 100%) under comparable flow conditions,^22^ CP∣CoP_L_ is three orders of magnitude more active (*i.e.*, TOF_CO_ = 0.5 h^−1^ after 24 h at 10% CO_2_*vs.* 413 h^−1^ after 4 h at 20% CO_2_, respectively). Without taking into account that CO_2_ is more soluble in organic solvents than in water (*e.g.* ∼180 mM in DMF *vs.* ∼33 mM in water at 25 °C), these differences in performance could be tentatively associated to the catalytic mechanism of CO_2_ reduction, which enables cobalt porphyrins to achieve higher TOF than rhenium bipyridine electrocatalysts.^22,37^

The electrooxidation of the liquid fraction or liquor, containing predominantly lignin and obtained from pre-treating lignocellulose (250 mg), was studied in a two-compartment electrochemical cell with a three-electrode setup. For this purpose, the anodic conditions were initially optimised using the lignin model substrate 1-(3,4-dimethoxyphenyl)-2-(2-methoxyphenoxy)propane-1,3-diol, which contains a β-O-4 linkage between two phenyl rings that mimics those ubiquitously found in lignin (see Fig. 1 and Fig. S7†).^9–11^

CP∣MWCNT anodes were fabricated by dropcasting a MWCNT suspension in ethanol (1.67 mg mL^−1^, 0.1 mL cm^−2^) containing Nafion 117 (2 vol% of a 5 wt% solution) to achieve a high surface area MWCNT layer with ∼20 μm thickness (see Fig. S8†). We found that MWCNT on hydrophilic carbon paper (CP∣MWCNT) acted as a suitable catalyst for the oxidation of the β-O-4 linkage in the model substrate (see Fig. S7 and ESI Note 2†). CP∣MWCNT (geometric surfaced area = 1 cm^2^) in the presence of 10 mM of lignin model substrate in 0.1 M Na_2_CO_3_ in 1 : 1 MeCN : H_2_O achieved high current densities (∼8 mA cm^−2^) at +1 V *vs.* Ag/AgCl (sat. KCl), while in absence of lignin, and under the same conditions, the current densities were lower (∼3 mA cm^−2^) (see Fig. S9 and S10†). MeCN was used to increase the solubility of the lignin model substrate and lignin. The generated oxidation product from the lignin model substrate during CA experiments, 3,4-dimethoxybenzaldehyde (3,4-MBA), was obtained in 36 ± 1% yield with a faradaic yield (FY) of 25 ± 1% assuming a two-electron oxidation (see Experimental details, Fig. S11 and Table S6†). The obtained 3,4-dimethoxybenzaldehyde was measured by ^1^H nuclear magnetic resonance (NMR) spectroscopy in CDCl_3_ with mesitylene as internal standard (see Fig. S12–S14†).

Having established the optimised conditions (see ESI Note 3, and Fig. S15–S20†) and the electrodes suitable for lignin oxidation and low concentration CO_2_ reduction, we aimed at coupling both redox half reactions in a single two-electrode electrolyser with the corresponding anolytes and catholytes separated by a bipolar membrane. Electrolysis was performed using a two-electrode setup (CP∣CoP_L_ as WE and CP∣MWCNT as CE) with an applied voltage (*U*_app_) of −3 V for 4 h. The anolyte comprised of 0.1 M Na_2_CO_3_ in a 1 : 1 MeCN : H_2_O solvent mix containing lignin (obtained from pre-treating 250 mg lignocellulose), and the catholyte had 0.1 M NaHCO_3_ in H_2_O. The catholyte was constantly purged at 9 mL min^−1^ with 20 vol% CO_2_ (balanced with N_2_). The gaseous products on the cathodic side were monitored by online GC.^38^

**Fig. 4:**
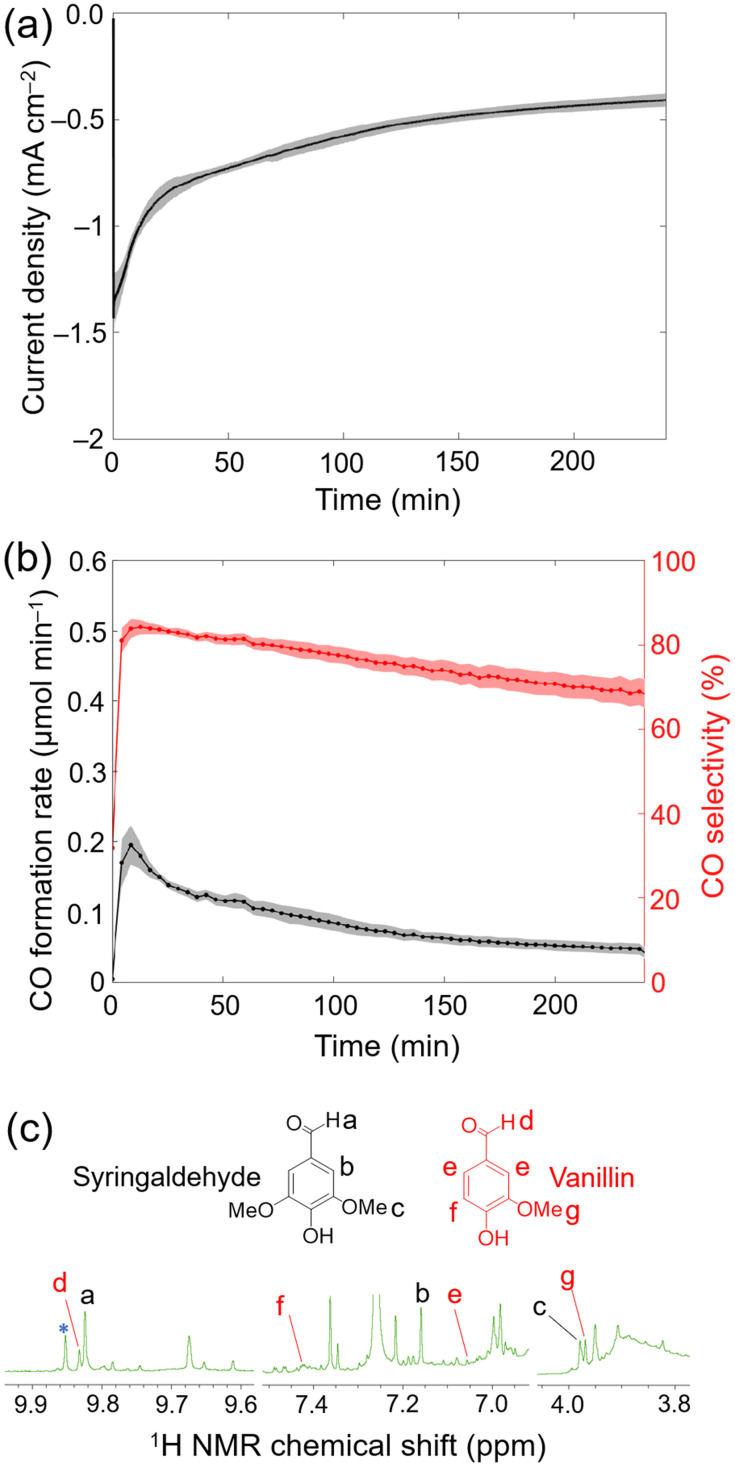
Electrolysis of 20 vol% CO_2_ gas flow with CP∣CoP_L_ at the cathode coupled to lignin conversion at the anode with CP∣MWCNT. (a) Current density as a function of time. (b) CO formation rate and selectivity during electrolysis at the cathode. (c) ^1^H NMR spectrum recorded in CDCl_3_ of the anolyte containing lignin after 4 h electrocatalysis showing the emergence of aldehydes and methoxy groups. Experiments were performed in triplicates, and the standard deviation are represented as shaded area (a, b). Reaction conditions: (a and b) Electrolysis (two electrode configuration) at *U*_app_ = −3 V. Anolyte: 0.1 M Na_2_CO_3_, lignin fraction obtained from pre-treating 250 mg lignocellulose in 1 : 1 MeCN : H_2_O; anode: CP∣MWCNT. Catholyte: 0.1 M NaHCO_3_ in H_2_O with a flow of 20 vol% CO_2_ (balanced with N_2_) 9 mL min^−1^; cathode: CP∣CoP_L_; bipolar membrane; room temperature. (c) The asterisk (*) highlights the additional aldehyde signal formed from lignin electrooxidation.

During 4 h electrolysis, the initial current density gradually decreased from approximately −1.4 mA cm^−2^ to around −0.4 mA cm^−2^ (Fig. S21†), and the initial maximum CO formation rate observed changed from ∼0.2 μmol_CO_ min^−1^ to ∼0.05 μmol_CO_ min^−1^ (Fig. 4a and b). After 4 h, 20.5 ± 0.2 μmol_CO_ cm^−2^ was produced along with 5.8 ± 0.3 μmol_H_2__ cm^−2^ (TON_CO_ = 707 ± 7 and TON_H_2__ = 200 ± 10) corresponding to a CO selectivity of 78 ± 2% and a FY_CO+H_2__ of 59 ± 6% (Table S5†). Despite of the decrease in current density and CO formation rates, the CO selectivity remained stable.^28^

After electrolysis, the anolyte was worked-up (see Experimental section for details) and the crude product was analysed by ^1^H NMR spectroscopy in CDCl_3_. Control experiments, where the lignin fraction was stirred in the electrolyte solution for 4 h without applied bias, led to the formation of a small background level of aldehydes. Importantly, under an applied potential more aromatic aldehyde signals were formed (Fig. S20† and Fig. 4c), which shows that aromatic monomer formation is promoted by electrooxidation. The observed ^1^H NMR signals corresponded to syringaldehyde and vanillin (see Fig. 1 and 4c), which are aromatics that can be formed from lignin.^8,10^ After 4 h CA, 0.14 ± 0.03 μmol and 0.31 ± 0.03 μmol of vanillin and syringaldehyde were detected, respectively. These yields equated to 0.62 g of vanillin and 1.65 g of syringaldehyde per kilogram of lignin. Compared with oxygen evolved from water oxidation, vanillin and syringaldehyde have potential as bio-derived monomers in the polymer industry.^40^ The observed moderate yields for vanillin and syringaldehyde could be attributed to the acidic degradation of reaction intermediates, *i.e.*, lignin-fragment oxidation decreased the local pH at the CP∣MWCNT anode surface below 11.5, and hence halt the formation of the targeted aromatics.^40^ In addition to the two identified aromatic compounds, other unidentified signals in the aromatic aldehyde (∼9.8 ppm) and methoxy regions (3.8–4.0 ppm) can be observed in the ^1^H NMR spectra (Fig. 4c).^40^

In comparison to CP∣MWCNT, the use of heterogeneous anodes for lignin oxidation based on metals/metal oxides, such as toxic Pb/PbO_2_, has been previously reported.^41–43^ For instance, these Pb/PbO_2_ anodes were able to generate different lignin-derived products, such as vanillin (5.83 g kg_lignin_^−1^) and syringaldehyde (9.30 g kg_lignin_^−1^), *via* electrooxidation/electrohydrogenation of bamboo-derived lignin when used with Cu cathodes in 1 M NaOH solution.^41^ Despite the difficulty to compare this previously reported system with our CP∣MWCNT∣∣CP∣CoP_L_ system due to the different experimental conditions (*i.e.*, electrode materials and surface, pH, temperature, substrate concentration, applied voltage and currents), the metal-free CP∣MWCNT anodes were able to generate yields of vanillin and syringaldehyde within the same order or one-order of magnitude lower than those reported for the metal/metal oxide based Pb/PbO_2_∣∣Cu system.

Previous work has shown that lignin valorisation can be coupled with hydrogen evolution or reduction of CO_2_ to formate using photoelectrochemical systems.^44,45^ In comparison, our work presents a unique and successful three-stage approach that shows that complete valorisation of lignocellulose is possible through the combination of fractionation and utilisation of photocatalysis and electrocatalysis to oxidise the resulting solid fraction to formate and liquid fraction to aromatics and reduce diluted CO_2_ and water to CO and H_2_. Although this system is a proof-of-concept demonstration, different factors would need consideration for practical implementation. These include optimisation of catalyst performance, scalability,^46,47^ integration of a carbon capture step,^48^ potential limitations such as catalyst stability, the engineering design of reactors^49,50^ for lignocellulose fractionation,^46^ photocatalysis^51^ and electrolysis,^52^ and the separation of products for further use.^53,54^

## Conclusions

We report a combined fractionation-photocatalysis-electrolysis process for the complete valorisation of lignocellulose and low-concentration CO_2_. Fractioning lignocellulosic wood provides sugar-based solid and lignin-based soluble fractions that are photo- and electrocatalytically converted with immobilised molecular cobalt(ii) catalysts to produce CO, formate, vanillin and syringaldehyde. The presented results set a precedent in integrating renewable processes for complete waste feedstock valorisation under ambient pressure and temperature. Moreover, our molecular hybrid systems were able to convert low CO_2_ concentrations (10–20 vol% CO_2_), which paves the way towards utilising CO_2_ concentrations nearing those found in flue gas (4–10 vol% CO_2_). However, to utilise actual flue gas additional CO_2_ purification steps are necessary to eliminate impurities,^55^ such as SO_2_, SO_3_, NO_x_, O_2_ and particulate matter depending on the type of fuel used. Learning from the use of low CO_2_ concentration also brings us a step closer towards operating directly from atmospheric CO_2_ (420 ppm).^20,56^

Hence, this work demonstrates a proof-of-concept strategy to valorise challenging multicomponent waste streams simultaneously through solar-driven and electrochemical redox processes. This work further highlights the potential of molecularly engineered hybrid materials^57^ in the valorisation of waste streams, which can be expanded beyond CO_2_ reduction in future developments to perform chemistry in a more sustainable and circular manner. As biomass and CO_2_ emerge as the most scalable and readily available sustainable carbon sources to defossilise the chemical industry,^58^ this work aims to inspire new approaches in their practical valorisation.

## Experimental section

### Solvents and materials

Acetonitrile (MeCN, Fisher Chemicals), D_2_O (Sigma Aldrich), d(+)-glucose (Fisher Chemicals), d(+)-cellobiose (Acros), Cellulase from *Trichoderma reesei* ATCC 26921 (Sigma Aldrich), sodium acetate trihydrate (Fisher Chemicals), hydrochloric acid (34–37% Fisher Chemicals), 1-(3,4-dimethoxyphenyl)-2-(2-methoxyphenoxy)propane-1,3-diol (lignin model substrate, FluoroChem), Vanillin (Thermo Scientific), syringaldehyde (Thermo Scientific), vanillic acid (Sigma Aldrich), syringic acid (Thermo Scientific), fufural (Sigma Aldrich), 2-methoxyphenoxyacetic acid (Alfa Aesar), 3,4-dimethoxybenzaldehyde (Acros), 4-hydroxybenzoic acid (Thermo Scientific), white birch (Merck), dioxane (Fisher Chemicals), formic acid (Fisher Chemicals), sulfuric acid (Fisher Chemicals), multiwalled carbon nanotubes (MWCNT 755117, Sigma Aldrich), carbon paper (Toray Paper 60, Fuel Cell Store), Nafion solution (5 wt% Sigma Aldrich), NaHCO_3_ (Merck), EtOAc (Fisher Chemicals), THF (Fisher Chemicals), Na_2_CO_3_ (Merck), MgSO_4_ (Fisher Chemicals) CDCl_3_ (Sigma Aldrich), 50 wt% H_2_SO_4_ (Fluka), Na_2_CO_3_ 64 mM and NaHCO_3_ 20 mM (ion chromatography eluent concentrate for Metrosep A Supp5; Sigma Aldrich) were purchased from commercial sources and used as received. MilliQ® grade H_2_O was used for all the experiments. TiO_2_ powder P25 (10–30 nm diameter; 50 m^2^ g^−1^) was obtained from Evonik; [Co(2,2′:6′,2′′-terpyridine-4′-phosphonic acid)_2_](BF_4_)_2_ (denoted as CotpyP)^35^ and CoP_L_ ^37^ were synthesised according to reported procedures. Reaction gases (CO_2_ and N_2_) were purchased from BOC. Ag/AgCl reference electrode was stored in a saturated NaCl solution (sat. NaCl; BasiMW-2030) and anion exchange membrane (Selemion, AGC Engineering), Nafion 117 membrane (Sigma Aldrich), bipolar membrane (Fumacep, Fuel Cell Store), were stored in MilliQ® grade H_2_O.

### Physical characterisation

^1^H NMR spectroscopy was recorded on a Bruker DPX 400 MHz spectrometer with the chemical shifts (*δ*) of the ^1^H NMR spectrum being referenced against the residual solvent signal (D_2_O: *δ* = 4.79 ppm, CDCl_3_: *δ* = 7.26 ppm). Scanning electron microscopy was performed on a TESCAN MIRA3 FEG-SEM instrument. Before measuring the samples, they were sputtered with 10 nm of platinum.

### Product quantification

Gaseous H_2_ and CO under static conditions were analysed by a Shimadzu Tracera GC-2010 Plus with a barrier discharge ionization detector. The GC-2010 Plus was equipped with a ShinCarbon micro ST column (0.53 mm diameter) kept at 40 °C using helium carrier gas. The response factors for the gases were determined by calibration with known amounts of H_2_ and CO. Typically, 50 μL of headspace gas from the photoreactor was injected using an air-tight syringe (Hamilton, GASTIGHT). Formate was analysed by ion chromatography (IC) on a Metrohm 882 compact IC plus chromatography system equipped with a Metrosep A Supp 5 – 150/4.0 column using an aqueous Na_2_CO_3_ (3.2 mM) and NaHCO_3_ (1 mM) solution as eluent. The response factor of formate was determined by calibration with known amounts of aqueous formate solutions. Sugar concentrations (glucose, cellobiose, xylose, mannose and arabinose) were determined by high performance liquid chromatography (HPLC) on a Waters Breeze system equipped with a refractive index detector and a Rezex 8% Ca^2+^ Monosaccharide 300 × 7.80 mm HPLC column using 2.5 mM H_2_SO_4_ as the eluent with a flow rate of 0.5 mL min^−1^ (at 75 °C). The response factors for the sugars were determined by calibration of aqueous sugar solutions with known amounts of sugar.

### Klason lignin and sugar content determination

Klason lignin and sugar determination was performed following a reported procedure.^6^ For the compositional analysis of white birch, the sample was first dried at 105 °C overnight followed by cooling to room temperature under static vacuum in a desiccator containing CuSO_4_ as drying agent. The dried solid was extracted three times with an ethanol : water mixture (4 : 1 v/v and 20 mL g^−1^) followed by water (20 mL g^−1^) by sonication for 30 min. The sample was then dried at 105 °C overnight followed by cooling to room temperature under static vacuum in a desiccator containing CuSO_4_ as a drying agent.

To determine the Klason lignin content and sugar concentration, to 250 mg of the solid (dried and extracted white birch or solid after white birch pre-treatment in dioxane/HCl/HCOOH) was added 3.75 mL of a 72 wt% H_2_SO_4_ at room temperature. The suspension was stirred periodically (every 15 min with a glass rod) for 2 h and 145 mL of MilliQ® grade H_2_O was added, followed by refluxing the suspension at 120 °C for 4 h. The suspension was filtered on a tared frit. The solid was dried at 105 °C overnight and the mass of the filtered solid was determined gravimetrically to determine Klason Lignin content. The filtrate was diluted with H_2_O to 250 mL and concentrated to 50 mL at 80 °C. The solution was then analysed by HPLC to determine the sugar content (glucose, xylose, mannose, galactose and arabinose).

To determine the Klason Lignin and sugar content of the liquor from white Birch pre-treatment in dioxane/HCl/HCOOH, the liquor obtained from pre-treating 500 mg white Birch was used following the same procedure as with dried and extracted white Birch.

### White Birch pre-treatment

Lignocellulose fractioning was performed following a reported procedure.^7^ To 500 mg of dried and extracted white birch was added dioxane (4.5 mL), conc. HCl (0.42 mL), formic acid (0.36 mL) and water (0.64 mL). The suspension was then stirred for 5 h at 80 °C and filtered and the residue was washed with dioxane until the solution became clear (solid/cellulose fraction). The combined filtrate was dried at 60 °C under vacuum and extracted with THF followed by drying at 40 °C to obtain a dark oil (liquid/lignin fraction).

### Enzyme hydrolysis of pre-treated white Birch

The obtained solid after treatment of white birch with dioxane/HCl/HCOOH was washed three times with H_2_O (20 mL g^−1^) to remove residual HCOOH and dried at 105 °C overnight. To the washed and dried solid fraction (150 mg) in an aqueous sodium acetate solution (50 mM, 3 mL) at pH 5 (adjusted by the addition of HCl) at 37 °C was added cellulase (15 mg in 0.75 mL) in a 50 mM aqueous sodium acetate solution at pH 5. The suspension was incubated at 37 °C for 24 h, followed by 15 min at 90 °C and filtration through a syringe filter (0.2 μm). The filtered solution was stored at −4 °C. The pre-treated cellulose fraction solution contained glucose (39.3 ± 4.7 mM) and cellobiose (8.7 ± 2.2 mM) as determined by HPLC. Control experiments with the recovered solid after treatment of white Birch with dioxane/HCl/HCOOH under the same condition in the absence of cellulase did not show the formation of glucose or cellobiose.

### CP∣MWCNT electrode preparation

A suspension containing MWCNT in ethanol (1.67 mg mL^−1^) and 2 vol% of a 5 wt% Nafion solution was sonicated for 15 minutes. The suspension was then dropcasted on a defined area of carbon paper (0.1 mL cm^−2^) that was masked with Teflon tape followed by overnight drying at room temperature. The electrode was then taped to a metal rod using a copper tape and the metal rod and copper tape were wrapped with Parafilm.

### CP∣CoP_L_ electrode preparation

A suspension containing MWCNT in DMF (3.16 mg mL^−1^) was sonicated for 10 min. Subsequently, this ink was diluted 25% by adding 0.4 mM CoP_L_ in DMF to achieve 2.37 mg MWCNT mL^−1^ and 0.1 mM CoP_L_. This ink was further sonicated for 10 min and then dropcasted on a defined area (masked with Teflon tape) of carbon paper (0.1 mL cm^−2^), followed by drying overnight at room temperature. The electrode was then taped to a metal rod using a copper tape and the metal rod and copper tape were wrapped with Parafilm.

### Photocatalytic experiments

In a typical experiment, to TiO_2_ (P25, 5 mg) was added 2 mL of MeCN and 1 mL of the solution obtained from cellulase pre-treatment in a glass photoreactor (7.74 mL total volume) equipped with a magnetic stir bar. 50 nmol of CotpyP (from a freshly prepared 2 mM solution in H_2_O; 0.025 mL) was added, and the photoreactor (3 mL solution with 4.74 mL headspace) was capped with a rubber septum and purged with CO_2_ (100 vol% or 20 vol% balanced with N_2_) controlled by mass flow controllers for 15 min at 15 mL min^−1^, followed by stirring for 15 min in the dark. The photoreactor (kept at 25 °C and stirred at 600 rpm) was then irradiated with a calibrated solar light simulator (Newport Oriel, 100 mW cm^−2^) equipped with an air mass 1.5 global (AM 1.5G) filter and a water filter to remove infrared radiation. The photocatalytic process was monitored by analysing the headspace after 6 and 24 h by gas chromatography to monitor H_2_ and CO formation. Formate in the solution (diluted in H_2_O (1 : 9 v : v photocatalysis solution : H_2_O)) was analysed at the end of the photocatalytic studies (after 24 h) by ion chromatography.

### CO_2_-to-CO conversion yield

Photocatalytic CO_2_-to-CO conversion yield (%) of TiO_2_∣CotpyP was calculated by dividing the mol of CO produced after 24 h by the mol of CO_2_ in the photoreactor headspace and multiplying the product by 100. The mol of CO_2_ was obtained using the ideal gas law equation (*pV* = *nRT*), where *p* is 1 atm, *V* is the volume of the reactor headspace in L multiplied by the molar fraction of CO_2_, *R* is the ideal gas constant (0.082 atm L mol^−1^ K^−1^) and *T* is 298.15 K.

### Isotopic labelling experiments

To a suspension of cellulose (300 mg) in a 50 mM aqueous sodium acetate solution (6 mL) at pH 5 (adjusted by the addition of HCl) at 37 °C was added cellulase (15 mg in 1.5 mL) in a 50 mM aqueous sodium acetate solution at pH 5. The suspension was incubated at 37 °C for 24 h, followed by 15 min at 90 °C and filtration through a syringe filter (0.2 μm). The filtered solution was stored at −4 °C. The pre-treated cellulose solution contained glucose (53 ± 2 mM) and cellobiose (26 ± 1 mM) as determined by HPLC. To a glass photoreactor vial (7.74 mL total volume) equipped with a magnetic stir bar was added 5 mg of TiO_2_ which was suspended in 2.95 mL of 2 : 1 v : v MeCN : pre-treated cellulose solution. The molecular catalyst CotpyP (0.025 mL 50 nmol, 2 mM in H_2_O) and 0.025 mL H_2_O (to reach 3 mL) was added and the photoreactor was capped with a rubber septum. The photoreactor was then degassed for one min (vacuum at 10^−2^ mbar) after which ^13^CO_2_ (1 bar) was introduced. The photoreactor (kept at 25 °C and stirred at 600 rpm) was then irradiated (AM 1.5G, 100 mW cm^−2^). The headspace was then transferred to an air-tight evacuated IR cell (10 cm path length, equipped with KBr windows) and the background (IR cell under vacuum) corrected IR spectrum was recorded to detect ^12^CO and ^13^CO.

### Flow CO_2_ electroreduction

Electrochemical experiments were performed on an Ivium Compactstat electrochemical analyser controlled by the Iviumsoft software. In a typical three-electrode setup experiment, a 0.1 M NaHCO_3_ solution in H_2_O was prepared and used as the anolyte and catholyte separated by a Nafion membrane. CA was performed with CP∣CoP_L_ as working electrode, Pt foil as counter electrode and Ag/AgCl as reference electrode. The electrochemical cell was capped with rubber septa and the catholyte was purged for 30 minutes with N_2_ (for screening electrocatalytic performance of different gas composition) or the desired gas composition (for CA at a given gas composition (20 or 100 vol%) for 4 h) at a flow rate of 20 mL min^−1^ controlled by mass flow controllers (Brooks) to remove oxygen. Afterwards the flow rate was reduced to 9 mL min^−1^ and a potential of −1.2 V *vs.* Ag/AgCl was applied. CA was run at −1.2 V *vs.* Ag/AgCl for 4 h at constant gas flow of 20 or 100 vol% or during screening different gas composition, the electrocatalytic activity was measured for 30 min under N_2_ followed by increasing the CO_2_ concentration to 10, 20, 50 and 100 vol% every 45 min. Electrochemical experiments were carried out at room temperature. The formed gaseous products (H_2_ and CO) were measured by online GC measurement (injection every 4.5 min) using an Shimadzu Tracera GC-2010 Plus gas chromatograph equipped a barrier discharge ionization detector.^38^ Calibration was performed by determining the response factor by flowing a calibration gas with known CO and H_2_ composition under the same condition (9 mL min^−1^).

### Electrocatalytic lignin model substrate & lignin oxidation

Electrochemical experiments were performed in three-electrode configuration on a PalmSens MultiEMStat^3+^ potentiostat. In a typical experiment, 0.1 M Na_2_CO_3_ solution in MeCN : H_2_O (1 : 1 vol%) was used as the electrolyte. To the electrolyte was added 0.01 M of the lignin model substrate or the lignin fraction from pre-treating 250 mg white birch in dioxane/HCl/H_2_O. The solution was then used as anolyte (8 mL) in electrocatalysis with 0.1 M Na_2_CO_3_ solution in MeCN : H_2_O (1 : 1 vol%), and separated from the catholyte by a Selemion anion exchange membrane, and the electrochemical cell was capped with rubber septa. Cyclic voltammetry (CV) or CA were performed with CP∣MWCNT as working electrode, Pt foil as counter electrode and Ag/AgCl (sat. KCl) as reference electrode. CV scans were run from −0.2 to 1.0 V *vs.* Ag/AgCl followed by a backwards scan to −0.2 V *vs.* Ag/AgCl with a scan rate of 50 mV s^−1^. CA experiments were performed for 4 h at 1.0 V *vs.* Ag/AgCl. Electrochemical experiments were carried out at room temperature. To analyse the products after electrocatalysis, the anolyte was acidified to a pH of 3 with 0.1 M HCl, extracted with EtOAc (3 × 5 mL), dried over MgSO_4_, filtered and dried at 40 °C under vacuum to obtain a light brown solid. The solid was further analysed by ^1^H NMR spectroscopy in CDCl_3_ with mesitylene as internal standard. Control experiments were performed without an applied potential and stirring the anolyte containing the lignin model substrate or lignin fraction for 4 h followed work up and analysis by ^1^H NMR spectroscopy in CDCl_3_.

### Electrocatalytic lignin oxidation coupled to CO_2_ reduction

Electrochemical experiments were performed in two-electrode configuration on an Ivium Compactstat electrochemical analyser controlled by the Iviumsoft software. In a typical experiment a 0.1 M Na_2_CO_3_ solution in MeCN : H_2_O (1 : 1 vol%) was prepared and used for the electrolyte. To the electrolyte was added 0.01 M of the lignin model substrate or the lignin fraction from pre-treating 250 mg white birch in dioxane/HCl/H_2_O. The solution was then used as anolyte (4 mL). A 0.1 M NaHCO_3_ solution in H_2_O was used as catholyte. The anolyte and catholyte were separated by a bipolar membrane. Electrolysis was performed with CP∣MWCNT and CP∣CoP_L_ as anode and cathode, respectively. The electrochemical H-type cell was capped with two rubber septa and the catholyte was purged for 30 minutes with 20 vol% CO_2_ (balanced by N_2_) at a flow rate of 20 mL min^−1^ controlled by mass flow controllers (Brooks) to remove oxygen. Afterwards the flow rate was reduced to 9 mL min^−1^ and a potential of *U*_app_ = −3 V was applied and run for 4 h. Electrochemical experiments were carried out at room temperature. The formed gaseous products (H_2_ and CO) on the cathode side were measured by online GC measurement (injection every 4.25 min) using Shimadzu Tracera GC-2010 Plus gas chromatograph equipped a barrier discharge ionization detector.^38^ Calibration was performed by determining the response factor by flowing a calibration gas with known CO and H_2_ composition under the same condition (9 mL min^−1^). To analyse the products, after electrocatalysis the anolyte was acidified to a pH of 3 with 0.1 M HCl, extracted with EtOAc (3 × 5 mL), dried over MgSO_4_, filtered and dried at 40 °C under vacuum to obtain a light brown solid. The solid was further analysed by ^1^H NMR in CDCl_3_ with mesitylene as internal standard. Control experiments were performed under the same conditions but without the lignin model substrate or lignin fraction dissolved in the anolyte.

### Single-pass CO_2_ conversion yield

The electrochemical single-pass CO_2_ conversion yield (%) of CP∣CoP_L_ was calculated by dividing the rate of CO formation (mol min^−1^) by the flow rate of CO_2_ (mol min^−1^) and multiplying the product by 100. The flow rate of CO_2_ was obtained by transforming mL min^−1^ to mol min^−1^ using the ideal gas law equation (*pV* = *nRT*), where *p* is 1 atm, *V* is the flow rate of CO_2_ in L min^−1^ (*e.g.*, 1.8 × 10^−3^ L min^−1^ for 20 vol% CO_2_), *R* is the ideal gas constant (0.082 atm L mol^−1^ K^−1^) and *T* is 298.15 K.

### Data analysis

Experiments were performed in triplicates and the results are represented with the mean (*x̄*) and standard deviation (*σ*_*x̄*_) expressed as *x̄* ± *σ*_*x̄*_ with
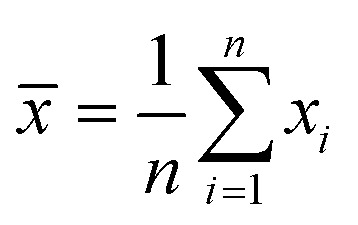

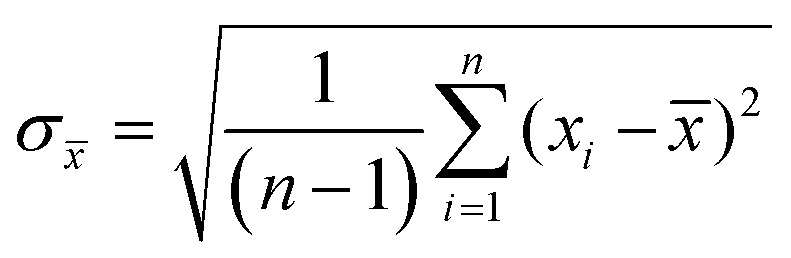
where *n* is the number of measurements and *x*_*i*_ the individually determined value.

## Conflicts of interest

There are no conflicts to declare.

## Supplementary Material

GC-025-D3GC03258B-s001
