# Supplementary material for: Valorisation of lignocellulose and low concentration CO2 using a fractionation–photocatalysis–electrolysis process
Source: Green Chem. 2023 Nov 13;25(24):10611–21. doi: 10.1039/d3gc03258b (PMC10711734; doi:10.1039/d3gc03258b)
Supplement: GC-025-D3GC03258B-s001 [file GC-025-D3GC03258B-s001.pdf]

Electronic Supplementary Material (ESI) for Green Chemistry.
This journal is © The Royal Society of Chemistry 2023

Electronic Supplementary Information

Valorisation of Lignocellulose and Low Concentration CO_2 using a Fractionation-Photocatalysis-Electrolysis Process

Santiago Rodríguez-Jiménez[1,2], *Erwin Lam*[1,2], *Subhajit Bhattacharjee*[1], *Erwin Reisner*[1]*

[1]Yusuf Hamied Department of Chemistry University of Cambridge, Lensfield Road, CB2 1EW, Cambridge, UK.

[2]These authors contributed equally.

*Corresponding author. E-mail: reisner@ch.cam.ac.uk

Table of Contents

Supplementary Notes ______ *S3*

Supplementary Tables ______ *S5*

Supplementary Figures ______ *S7*

Supplementary Notes

Supplementary Note 1: Chronoamperometry of CP|CoP$_L$ under constant CO_2 gas flow. Chronoamperometry (CA) was performed under a constant gas flow of 100 or 20 vol% CO_2 at –1.2 V vs. Ag/AgCl (sat. KCl) with CP|**CoP$_L$** for 4 h. Under 100 vol% CO_2, a total of 131±4 μmol_{CO} cm^{-2} (TON_{CO} = 4517±138) was formed after 4 h with a CO selectivity of (94±1)% and FY_{CO} of (95±2)% (Figure S2 and Table S5). Under 20 vol% CO_2, a total of 47.9±0.7 μmol_{CO} cm^{-2} (TON_{CO} = 1652±24) was formed after 4 h with a CO selectivity of (85±1)% and FY_{CO} of (70±2)% (Figure S3 and Table S5). The results highlight the catalyst stability and high CO selectivity and activity of CP|**CoP$_L$** at 20 vol% with respect to 100 vol% CO_2.

Supplementary Note 2: Lignin model substrate oxidation. CA was performed at +1 V vs. Ag/AgCl for 4 h in a three-electrode configuration using CP|MWCNT as working electrode (WE), Pt foil as counter electrode (CE) and Ag/AgCl as reference electrode (RE) separated by an anion exchange membrane (Figure S5). During CA, the current density initially reached ~6.5 mA cm^{-2} dropping to ~1.0 mA cm^{-2} over the course of the reaction with the anolyte turning from colorless to brown (Figure S11). After CA and following work up of the anolyte (see details in the Experimental Details), the products were analyzed by ^{1}H nuclear magnetic resonance (NMR) spectroscopy in $CDCl_3$ with mesitylene as internal standard (Figures S12, S13 and S17). The oxidation product detected was 3,4-dimethoxybenzaldehyde (3,4-MBA) with a concentration of 3.6±0.1 mM (Figure S13 and Table S6). The amount of 3,4-MBA corresponds to a yield of (36±1)% and a faradaic yield ($FY_{3,4\text{-MBA}}$) of 25±1% assuming a 2-electron oxidation process. Blank experiments without any applied potential showed no 3,4-MBA formation (Figure S14) due to the absence of any oxidation reaction. Thus, the fabricated CP|MWCNT electrodes are suitable to oxidize the lignin model substrate and generate monomeric aromatics.

Supplementary Note 3: Lignin model substrate oxidation coupled to CO_2 reduction. Electrolysis was performed using a two-electrode setup (CP|**CoP$_L$** as WE and CP|MWCNT as CE) with an applied potential of U_{app} = –3 V for 4 h in 0.1 M Na_2CO_3 in a 1:1 MeCN:H_2O solvent mix containing 0.01 M lignin model substrate (1-(3,4-dimethoxyphenyl)-2-(2-methoxyphenoxy)propane-1,3-diol) as anolyte and 0.1 M $NaHCO_3$ in H_2O as catholyte. Anode and cathode were separated by a bipolar membrane. The catholyte was constantly purged with 20 vol% CO_2 (balanced with N_2 at 9 mL min^{-1}). The gaseous products on the cathode side were monitored by online GC (Figure S6).

The current density remained relatively constant at –1.0 mA cm^{-2} during the 4 h of electrolysis (Figure S15). The CO formation rate reached a maximum of ~0.2 μmol_{CO} min^{-1} CO after around 40 min with the selectivity being above ~60% (Figure S15). The accumulated products after 4 h were 37.7±0.6

μmol_{CO} cm^{-2} and 18.3±0.4 μmol_{H_2} cm^{-2} corresponding to a CO selectivity of (67±8)% with a FY_{CO} of (58±8)% and a FY_{H_2} of (29±7)% (Figure S16 and Table S5). On the anodic side, 9.6±0.1 $\mu mol_{3,4\text{-}MBA}$ cm^{-2} was formed corresponding to a $FY_{3,4\text{-}MBA}$ of (24±4)% assuming a 2-electron oxidation process (Figure S16 and Table S6). Thus, both isolated redox half-reactions can be coupled together to simultaneously reduce low concentration CO_2 and oxidize the lignin model substrate. Control experiments were performed under the same conditions where the anolyte did not contain the lignin model substrate where most likely oxygen evolution predominantly occurs. Lower amounts of CO (29.4±0.3 μmol_{CO} cm^{-2}) and H_2 (6.6±0.2 μmol_{H_2} cm^{-2}) were formed supporting the promotional effect of coupling both half-reactions together to improve the overall productivity on the cathode side, while at the same time generating products beyond the thermodynamically more challenging O_2 evolution at the anode side (Figure S4 and Table S5).

Supplementary Tables

Table S1. Compositional analysis of white birch, the solid and liquid fraction after white birch treatment with Dioxane/HCl/HCOOH and solid fraction incubation in cellulase.

Entry	Sample	Glucose [wt%]	Xylose [wt%]	Mannose [wt%]	Arabinose [wt%]	Lignin [wt%]
1	White birch	34.7±1.6	20.3±1.0	1.8±0.1	0.9±0.1	17.6±1.4
2	Solid Fraction	62.9±1.9	6.7±1.6	>0.1	>0.1	5.4±1.2
3	Cellulase Treated Solid Fraction (Solution)	21.2±0.8[a] 8.6±1.3[b]	n.d.	n.d.	n.d.	n.d.
4	Liquid Fraction	2.4±0.6	6.9±1.3[c]	1.3±0.3	2.5±0.7	41.2±2.1

[a] as glucose. [b] as cellobiose. [c] xylose is partially converted to furfural (0.95±0.34 µmol detected by ^{1}H NMR after electrolysis). n.d. stands for not detected.

Table S2. Compositional analysis of white birch, the solid and liquid fraction after white birch treatment with Dioxane/HCl/HCOOH and solid fraction incubation in cellulase with respect to the overall composition of white birch.

Entry	Sample	Glucose [wt%]	Xylose [wt%]	Mannose [wt%]	Arabinose [wt%]	Lignin [wt%]
1	White birch	34.7±1.6	20.3±1.0	1.8±0.1	0.9±0.1	17.6±1.4
2	Solid Fraction	30.4±0.9	3.2±0.8	>0.1	>0.1	2.5±0.6
3	Cellulase Treated Solid Fraction (Solution)	10.2±0.4[a] 4.2±0.6[b]	n.d.	n.d.	n.d.	n.d.
4	Liquid Fraction	0.8±0.1	2.2±0.3	0.4±0.1	0.8±0.2	13.7±0.6

[a] as glucose. [b] as cellobiose. n.d. stands for not detected.

Table S3. Mass fraction recovered from the solid and liquid (or liquor) fraction after white birch treatment with Dioxane/HCl/HCOOH.

Entry	Sample	Solid Fraction [wt%]	Liquid Fraction [wt%]
1	White birch	48.3±0.8	38.9±0.5

Table S4. Photocatalytic H_2, CO and formate formation using TiO_2|**CotpyP** under 100 and 20 vol% CO_2 concentration (balanced with N_2). Reaction conditions: 50 nmol **CotpyP** and 5 mg TiO_2 (P25) in 3 mL of a 2:1 MeCN:aqueous sodium acetate (50mM, pH 5) solution with cellulase pre-treated lignin solid fraction, 25 °C 100 mW cm^{-2} AM 1.5G. Headspace volume = 4.74 mL.

Entry	Gas Composition	Time [h]	H_2 [μmol]	TON_{H2}	CO [μmol]	TON_{CO}	$HCOO^-$ [μmol]	Selectivity CO [%][a]
1	100 vol% CO_2	6	0.09±0.04	1.8	0.07±0.02	1.4	-	44±13
		24	0.55±0.04	11	0.35±0.02	7	0.77±0.02	39±2
2	20 vol% CO_2	6	0.18±0.06	3.6	0.01±0.01	0.2	-	5±5
		24	0.71±0.14	14.2	0.14±0.01	2.8	0.74±0.06	16±5

[a] Selectivity CO only accounts for the products generated by **CotpyP**, i.e. CO and H_2. TON stands for turnover number and is calculated by dividing mol of product per mol of catalyst.

Table S5. Electrochemical results from chronoamperometry of CO_2 electroreduction with CP|**CoP$_L$**. Reaction conditions: (Entry 1, 2, three-electrode setup, red) Chronoamperometry: 0.1 M $NaHCO_3$ in H_2O; –1.2 V vs. Ag/AgCl; 4 h WE: CP|**CoP$_L$**, CE: Pt foil, RE: Ag/AgCl; Flow rate 9 mL min^{-1}; 20 or 100 vol% CO_2. (Entry 3-5, two-electrode setup, blue) Electrolysis at U_{app} = –3 V. Anolyte: 0.1 M Na_2CO_3, 0.01 M lignin model substrate or lignin fraction obtained from pre-treating 250 mg Lignocellulose in 1:1 MeCN:H_2O; Anode: CP|MWCNT. Catholyte: 0.1 M $NaHCO_3$ in H_2O Flow of 20 vol% CO_2 (balanced with N_2) 9 mL min^{-1}; Cathode: CP|**CoP$_L$**.

Entry	Sample	CO [μmol cm^{-2}]	H_2 [μmol cm^{-2}]	Selectivity CO [%]	Charge [|C|]	FY_{CO} [%]	FY_{H2} [%]	FY_{CO+H2} [%]
1	100 vol% CO_2	130.7±4.7	7.8±0.6	94±1	26.5±0.8	95±2	6±1	101±2
2	20 vol% CO_2	48.9±0.7	8.2±0.5	85±1	13.3±0.5	70±2	12±1	82±2
3	20 vol% CO_2[a]	29.4±3.0	6.6±0.3	81±1	8.9±0.1	64±6	14±1	78±7
4	20 vol% CO_2 Lignin Model Substrate	37.7±5.7	18.3±3.7	67±8	12.5±0.8	58±8	29±7	87±3
5	20 vol% CO_2 Lignin Solid Fraction	20.5±2.4	5.8±0.3	78±2	8.6±0.4	46±5	13±1	59±6

[a] Electrolysis performed with CP|MWCNT without any lignin model substrate in 0.1 M Na_2CO_3 in 1:1 MeCN:H_2O

Table S6. Electrocatalytic results from chronoamperometry of lignin model substrate with CP|MWCNT to form 3,4-dimethoxybenzaldehyde. Reaction conditions: (Entry 1, three-electrode setup, red) Chronoamperometry: 0.1 M Na_2CO_3, 0.01 M lignin model substrate in 1:1 MeCN:H_2O; E_{appl} = +1 V vs. Ag/AgCl; 4 h WE: CP|MWCNT, CE: Pt foil, RE: Ag/AgCl. (Entry 2, two-electrode setup, blue) Electrolysis at U_{app} = –3 V. Anolyte: 0.1 M Na_2CO_3, 0.01 M lignin model substrate in 1:1 MeCN:H_2O; Anode: CP|MWCNT. Catholyte: 0.1 M $NaHCO_3$ in H_2O Flow of 20 vol% CO_2 (balanced with N_2) 9 mL min^{-1}; Cathode: CP|**CoP$_L$**.

Entry	Sample	3,4-MBA [μmol cm^{-2}]	Yield [%]	Charge [|C|]	$FY_{3,4\text{-}MBA}$ [%]
1	Lignin Model Substrate	28.5±0.9	36±1	22.1±0.7	25±1
2	20 vol% CO_2 Lignin Model Substrate	9.6±1.6	24±4	12.5±0.8	15±3

Supplementary Figures

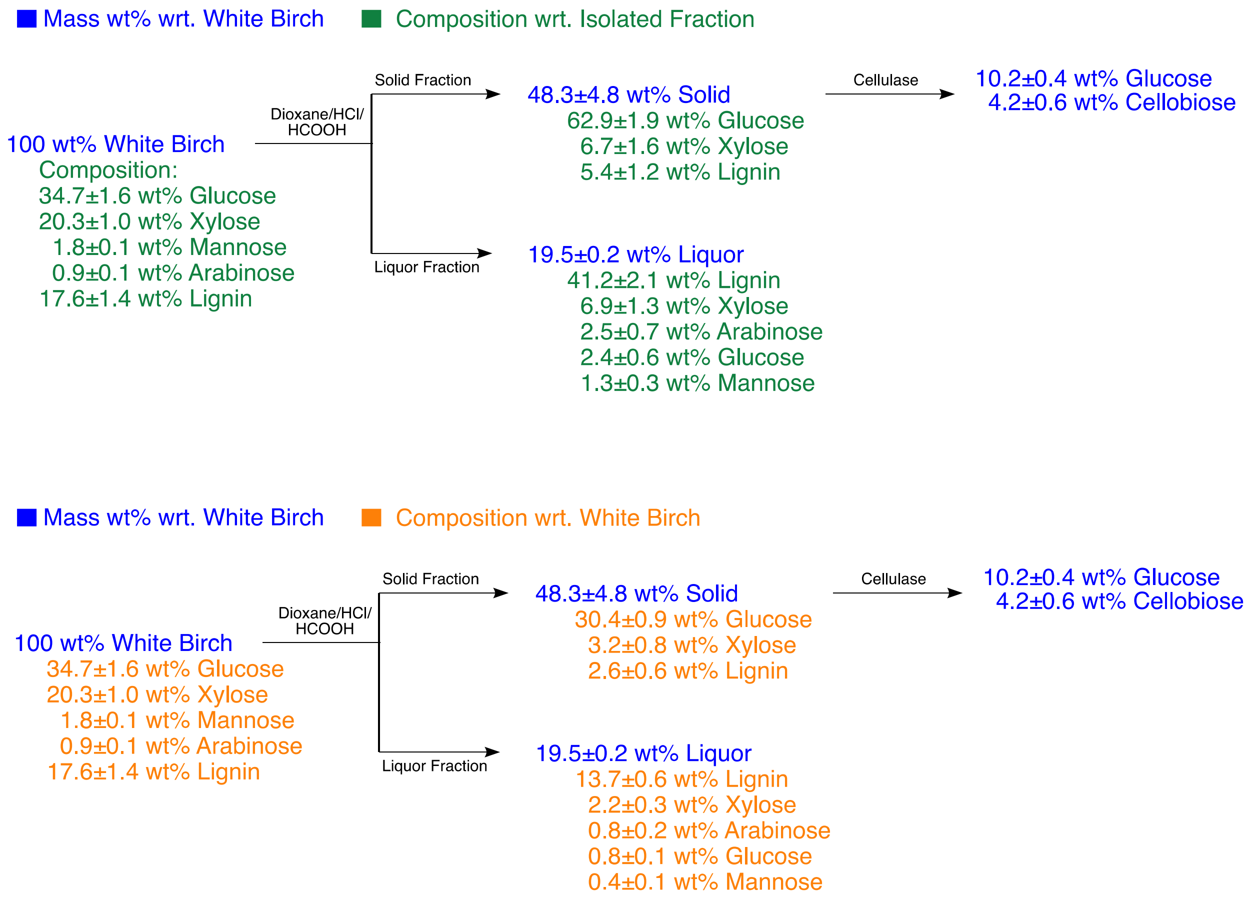

Figure S1. Fractioning of lignocellulose into solid and liquid fractions. Fractioning of Lignocellulose (white Birch) into a predominant cellulose and lignin containing fractions, and their composition with respect to (wrt) the isolated fractions (top) and white birch (bottom). Cellulose based solid fraction was further incubated with cellulase to generate a soluble sugar (glucose and cellobiose) solution.

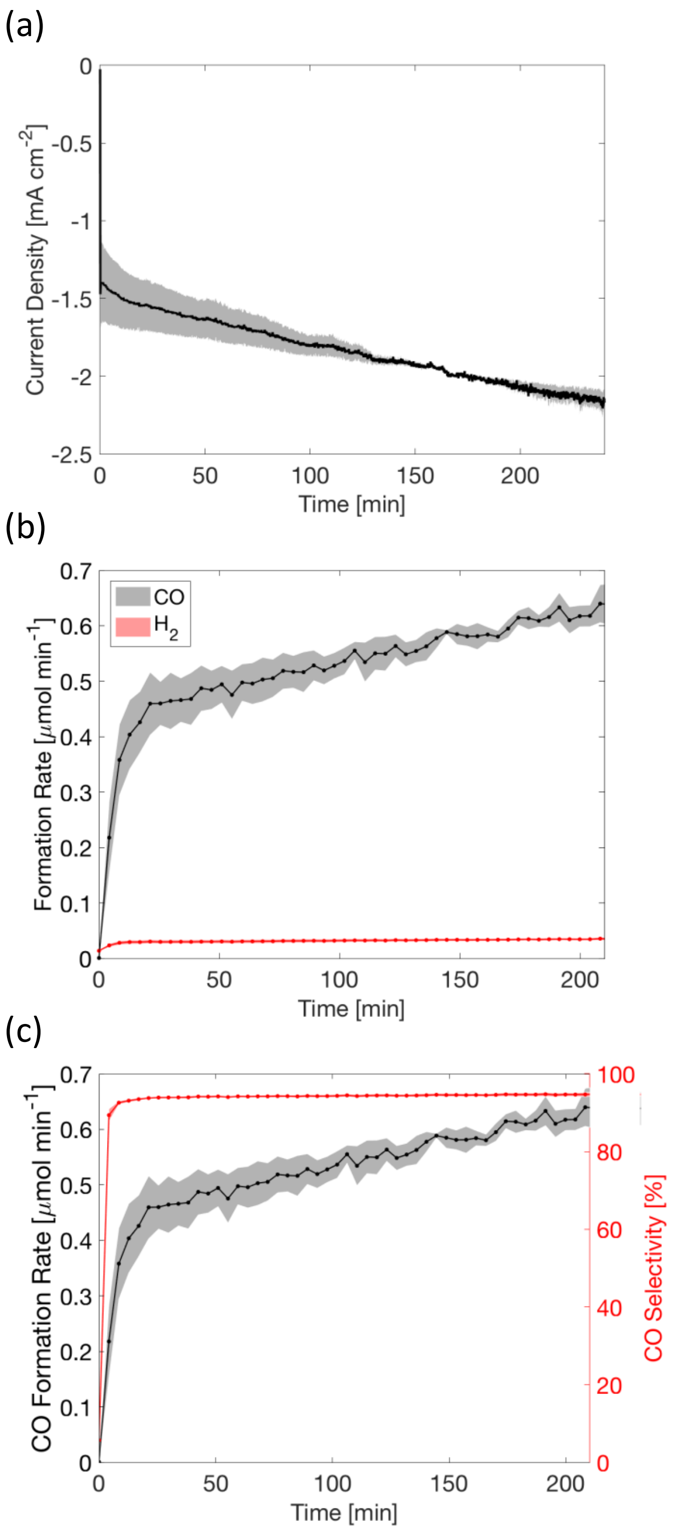

Figure S2. CO_2 chronoamperometry under 100 vol% CO_2 using a three-electrode setup. Chronoamperometry of CO_2 electroreduction under 100 vol% CO_2 gas flow with CP|**CoP_L**. (a) Current density with time. (b) CO and H_2 formation rate obtained from online GC. (c) CO formation rate and selectivity. The shaded area represents the standard deviation. Reaction conditions: Chronoamperometry: 0.1 M $NaHCO_3$ in H_2O; –1.2 V vs. Ag/AgCl; 4 h WE: CP|**CoP_L**, CE: Pt foil, RE: Ag/AgCl; Flow rate 9 mL min^{-1}; 100 vol% CO_2, room temperature.

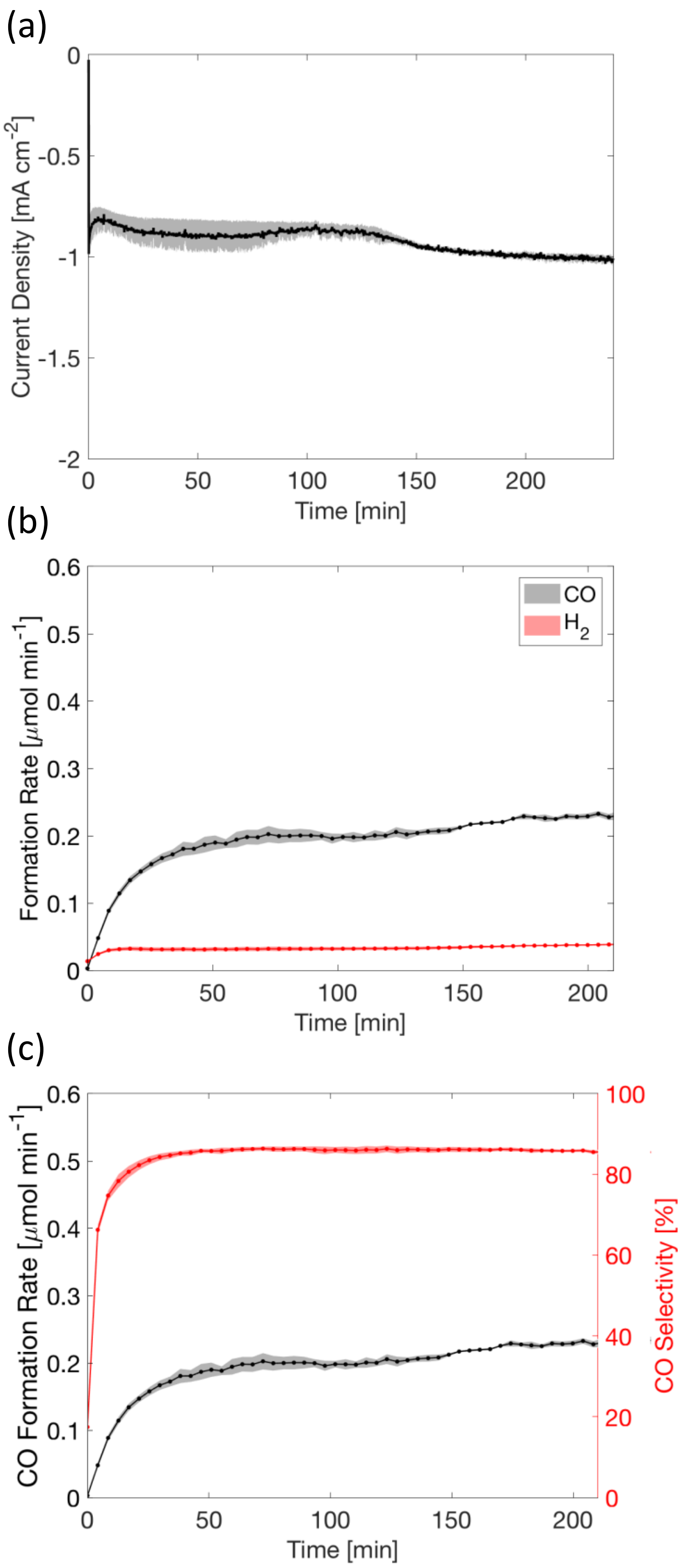

Figure S3. CO_2 chronoamperometry under 20 vol% CO_2 using a three-electrode setup. Chronoamperometry of CO_2 electroreduction under 20 vol% CO_2 gas flow with CP|**CoP$_L$**. (a) Current density with time. (b) CO and H_2 formation rate obtained from online GC. (c) CO formation rate and selectivity. The shaded area represents the standard deviation. Reaction conditions: Chronoamperometry: 0.1 M $NaHCO_3$ in H_2O; –1.2 V vs. Ag/AgCl; 4 h WE: CP|**CoP$_L$**, CE: Pt foil, RE: Ag/AgCl; Flow rate 9 mL min^{-1}; 20 vol% CO_2, room temperature.

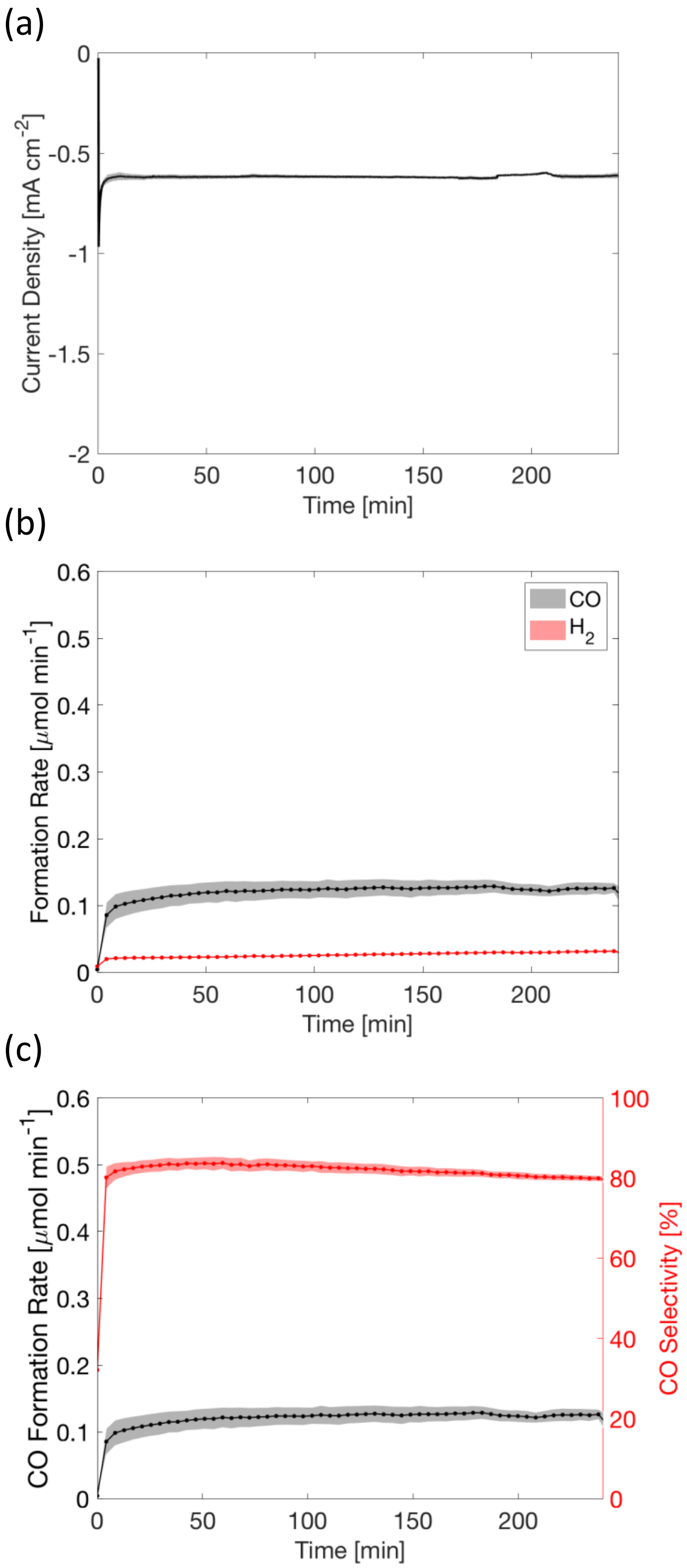

Figure S4. CO_2 electrolysis under 20 vol% CO_2 using a two-electrode setup (no lignin). CO_2 electroreduction under 20 vol% CO_2 gas flow with CP|**CoP$_L$** with CP|MWCNT as anode. (a) Current density with time. (b) CO and H_2 formation rate obtained from online GC. (c) CO formation rate and selectivity. The shaded area represents the standard deviation. Reaction conditions: Electrolysis at U_{app} = –3 V. Anolyte: 0.1 M Na_2CO_3 in 1:1 MeCN:H_2O; Anode: CP|MWCNT. Catholyte: 0.1 M $NaHCO_3$ in H_2O Flow of 20 vol% CO_2 (balanced with N_2) 9 mL min^{-1}; Cathode: CP|**CoP$_L$**, room temperature**.**

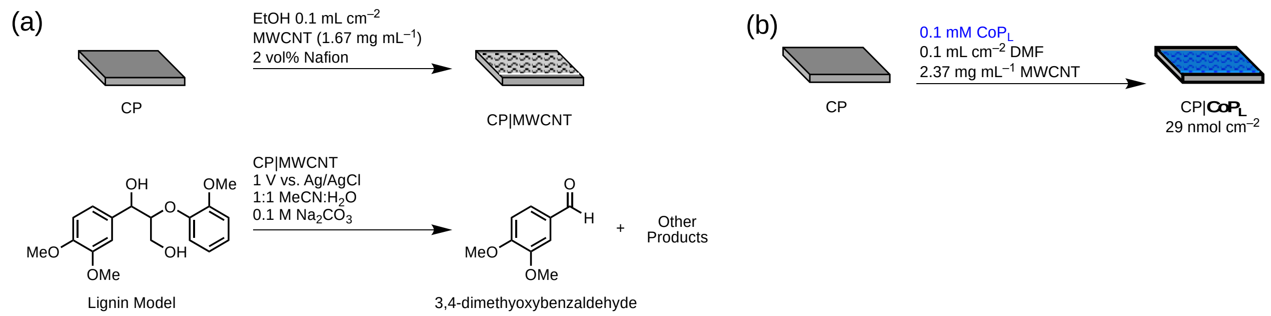

Figure S5. Electrode preparation. (a) Preparation of CP|MWCNT electrode and electrocatalytic lignin model substrate oxidation. (b) Preparation of CP|**CoP$_L$** electrode for CO_2 electroreduction to CO.

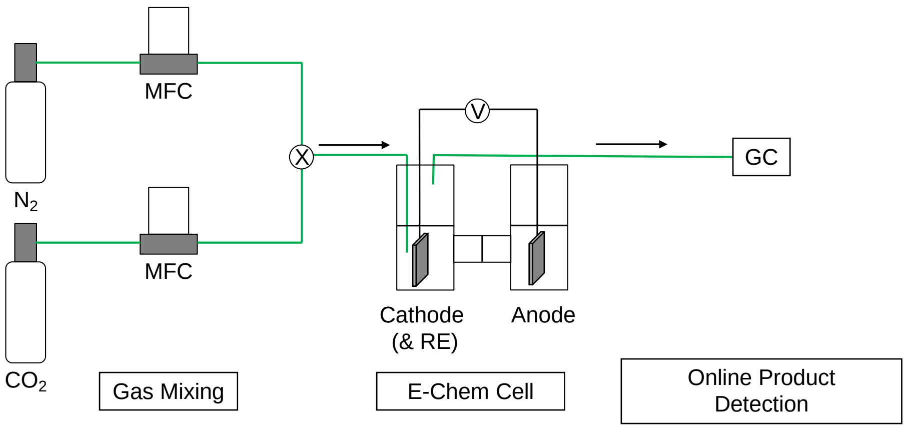

Figure S6. Flow setup for variable concentration CO_2 electrocatalysis. Scheme for CO_2 electrocatalysis under flow by controlling the gas composition with mass flow controllers purging the gas through the anolyte followed by online-GC analysis to detect H_2 and CO.[1]

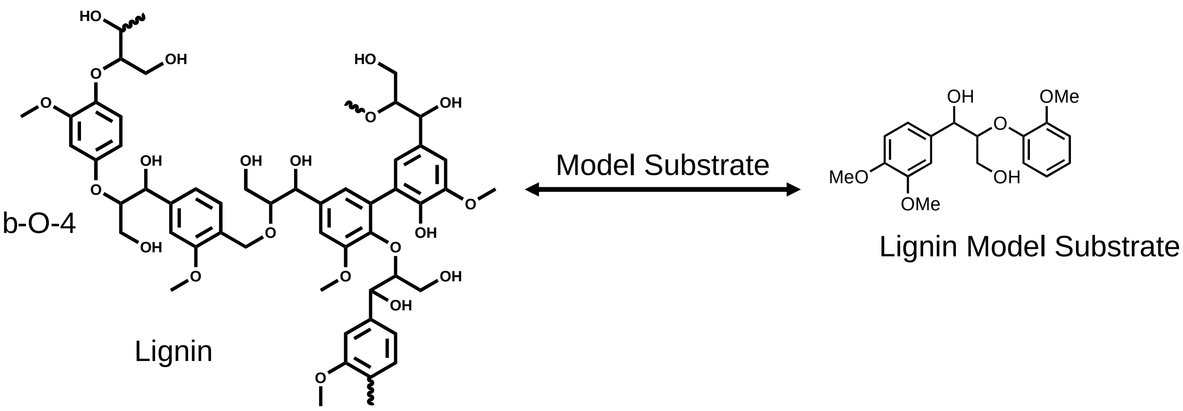

Figure S7. Structures of lignin polymer and lignin model substrate. Lignin polymer and its most common β-O-4 linkage to connect aromatic monomers and structure of lignin model substrate 1-(3,4-dimethoxyphenyl)-2-(2-methoxyphenoxy-propane-1,3-diol (i.e. 3,4-MBA) as dimer with the same β-O-4 linkage.

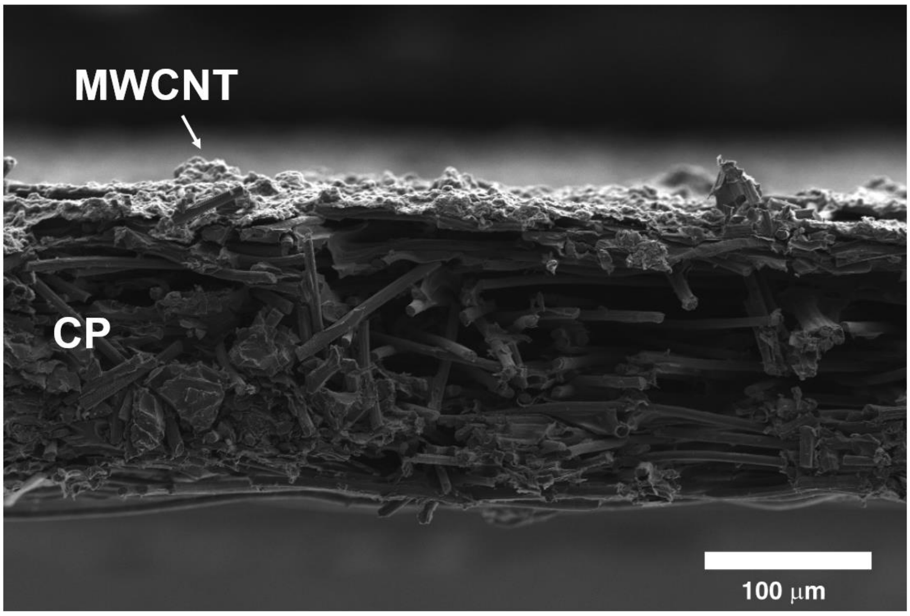

Figure S8. Scanning electron microscopy of anode. Scanning electron microscopy image of the cross section of CP|MWCNT.

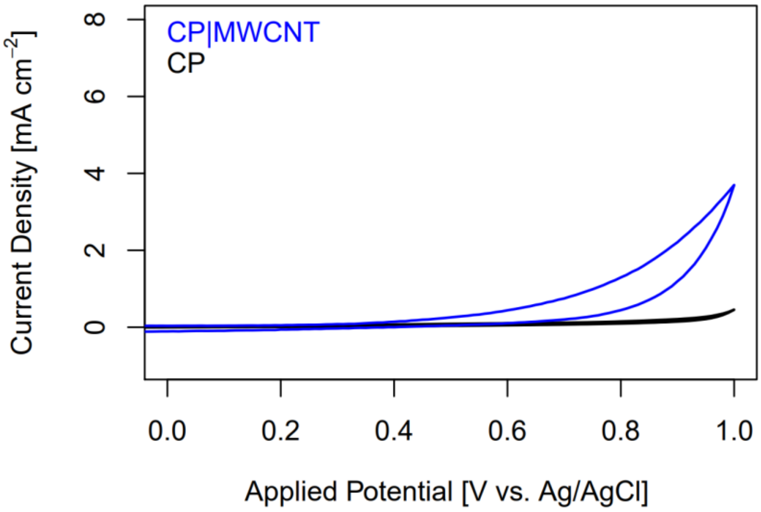

Figure S9. Optimisation of electrooxidation conditions (blank). Cyclic voltammetry of CP|MWCNT and CP. Reaction conditions: 0.1 M Na_2CO_3 in 1:1 MeCN:H_2O; –0.2 to +1 V vs. Ag/AgCl forward and backward scan; 50 mV s^{-1} CE: Pt foil, RE: Ag/AgCl, room temperature.

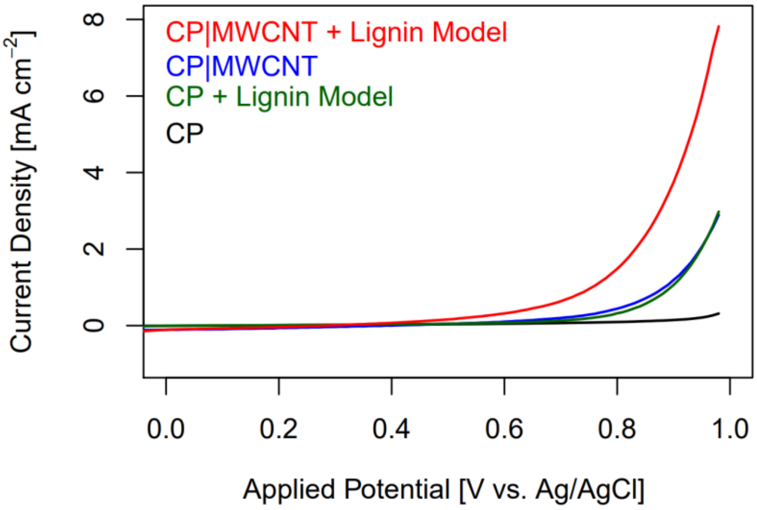

Figure S10. Optimisation of electrooxidation conditions. Cyclic voltammetry of CP|MWCNT and CP with and without the lignin model substrate (0.01 M). Reaction conditions: 0.1 M Na_2CO_3 in 1:1 MeCN:H_2O; –0.2 to +1 V vs. Ag/AgCl forward and backward scan; 50 mV s^{-1} CE: Pt foil, RE: Ag/AgCl, room temperature.

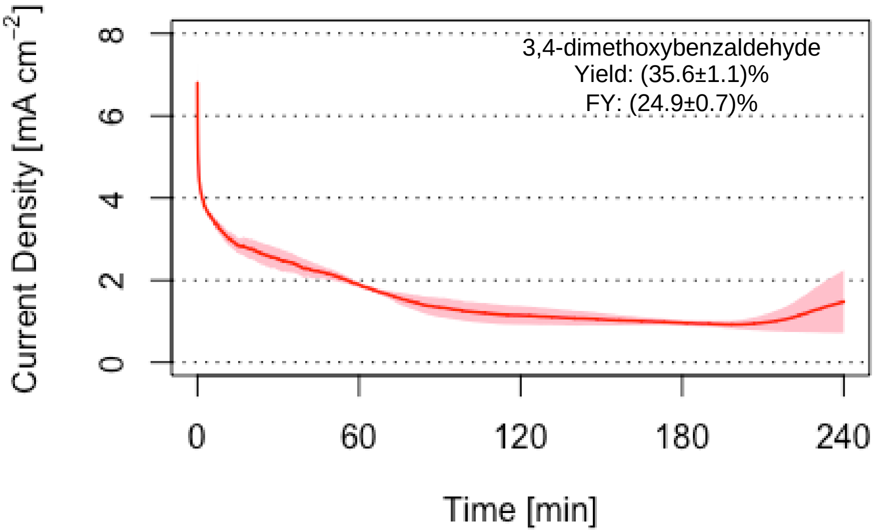

Figure S11. Chronoamperometry of optimised anode with lignin substrate model in three-electrode setup. Chronoamperometry trace of lignin model substrate with CP|MWCNT to form 3,4-dimethoxybenzaldehyde. The shaded area represents the standard deviation. Reaction conditions: CA: 0.1 M Na_2CO_3, 0.01 M Lignin Model in 1:1 MeCN:H_2O; +1 V vs. Ag/AgCl; 4 h WE: CP|MWCNT, CE: Pt foil, RE: Ag/AgCl, room temperature.

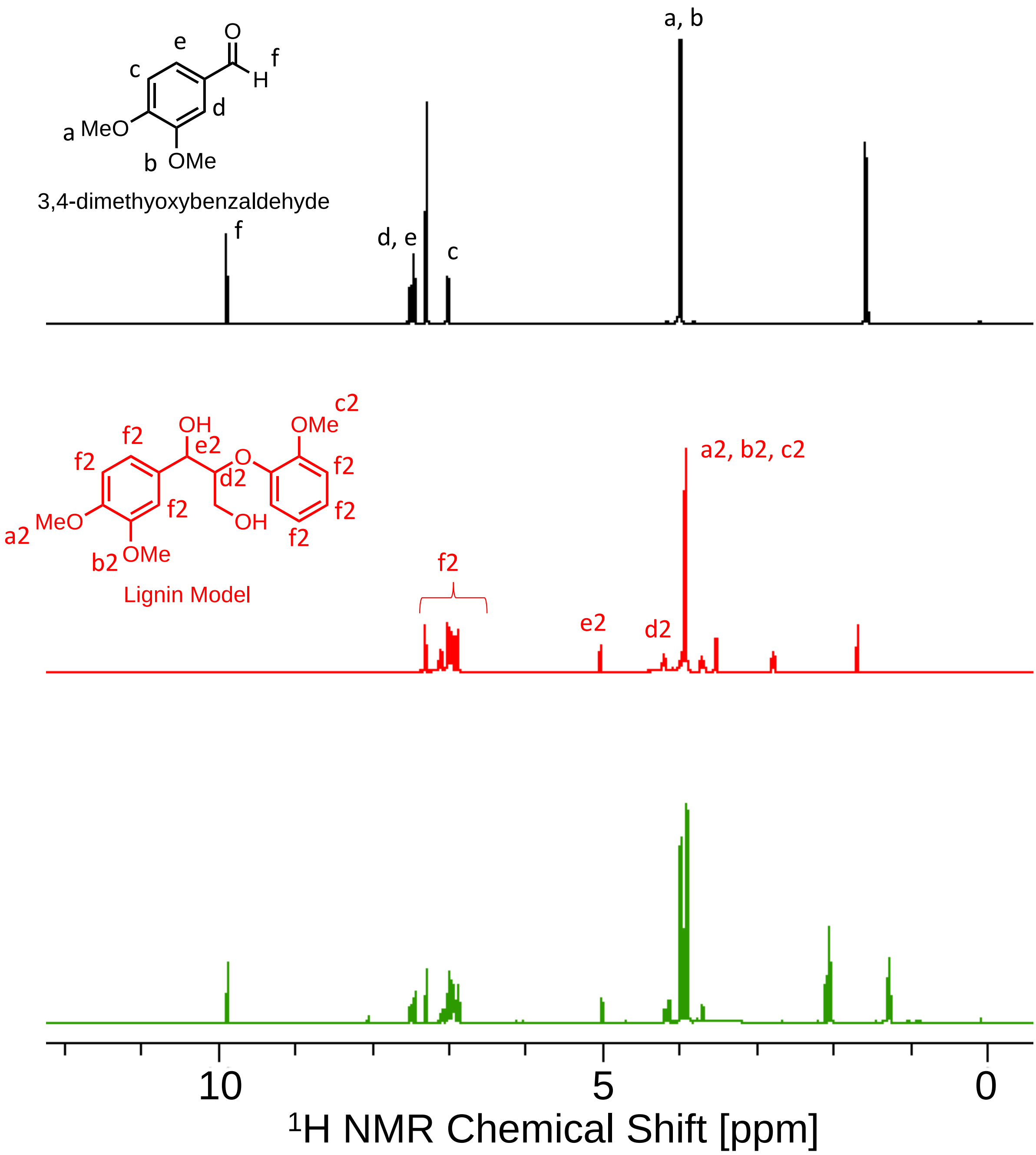

Figure S12. ¹H NMR of electrooxidised lignin model substrate in three-electrode setup. ¹H NMR of lignin model substrate after 4 h of chronoamperometry; ¹H NMR recorded in $CDCl_3$ with 3,4-dimethoxybenzaldehyde and the lignin model substrate as reference. Reaction conditions: Chronoamperometry: 0.1 M Na_2CO_3, 0.01 M lignin model substrate in 1:1 MeCN:H_2O; +1 V vs. Ag/AgCl; 4 h WE: CP|MWCNT, CE: Pt foil, RE: Ag/AgCl, room temperature.

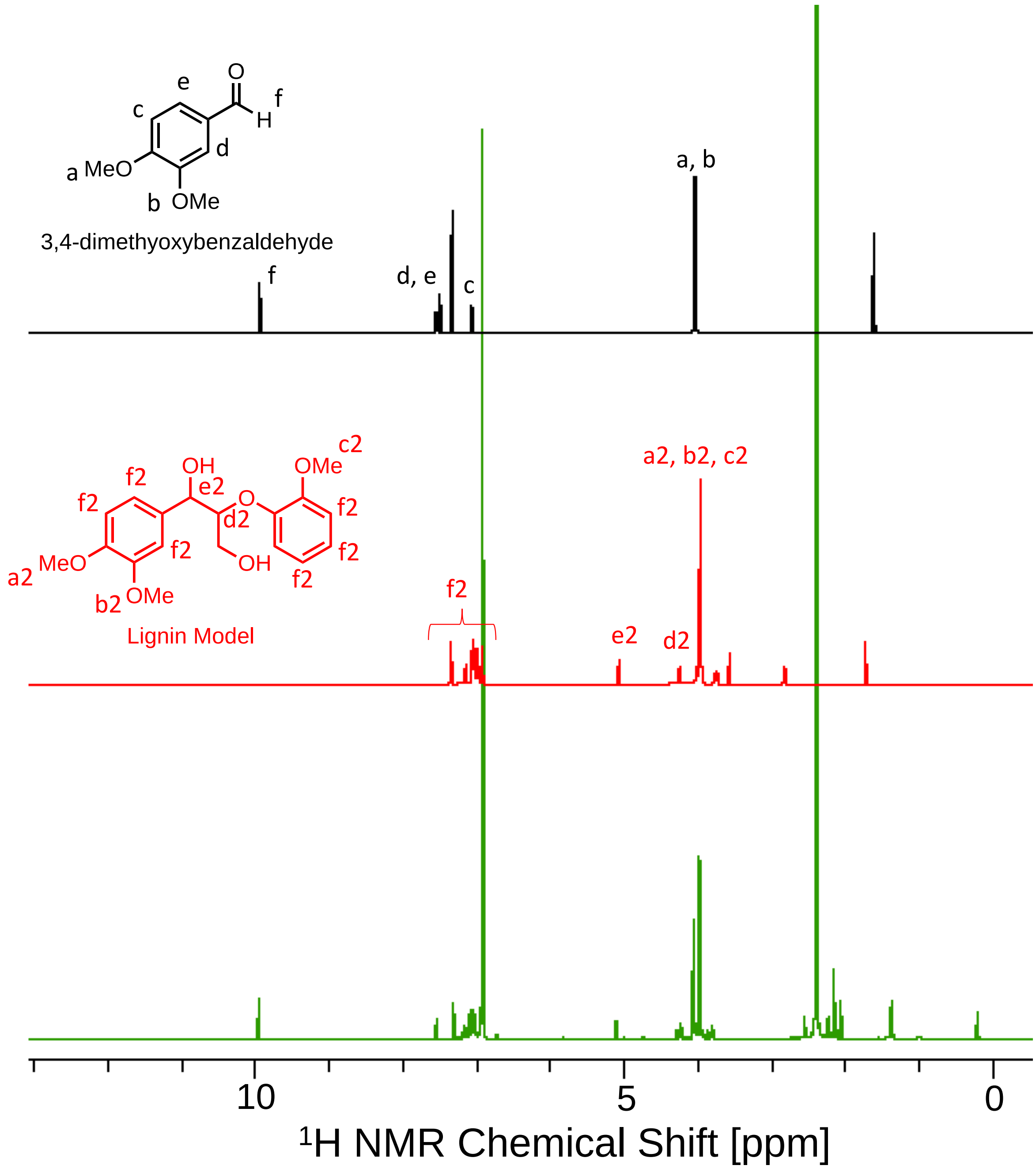

Figure S13. ^{1}H NMR of electrooxidised lignin model substrate in three-electrode setup. ^{1}H NMR of lignin model substrate after 4 h of chronoamperometry; ^{1}H NMR recorded in $CDCl_3$ with mesitylene as internal standard and 3,4-dimethoxybenzaldehyde and the lignin model substrate as reference spectra. Reaction conditions: Chronoamperometry: 0.1 M Na_2CO_3, 0.01 M lignin model substrate in 1:1 MeCN:H_2O; +1 V vs. Ag/AgCl; 4 h WE: CP|MWCNT, CE: Pt foil, RE: Ag/AgCl, room temperature.

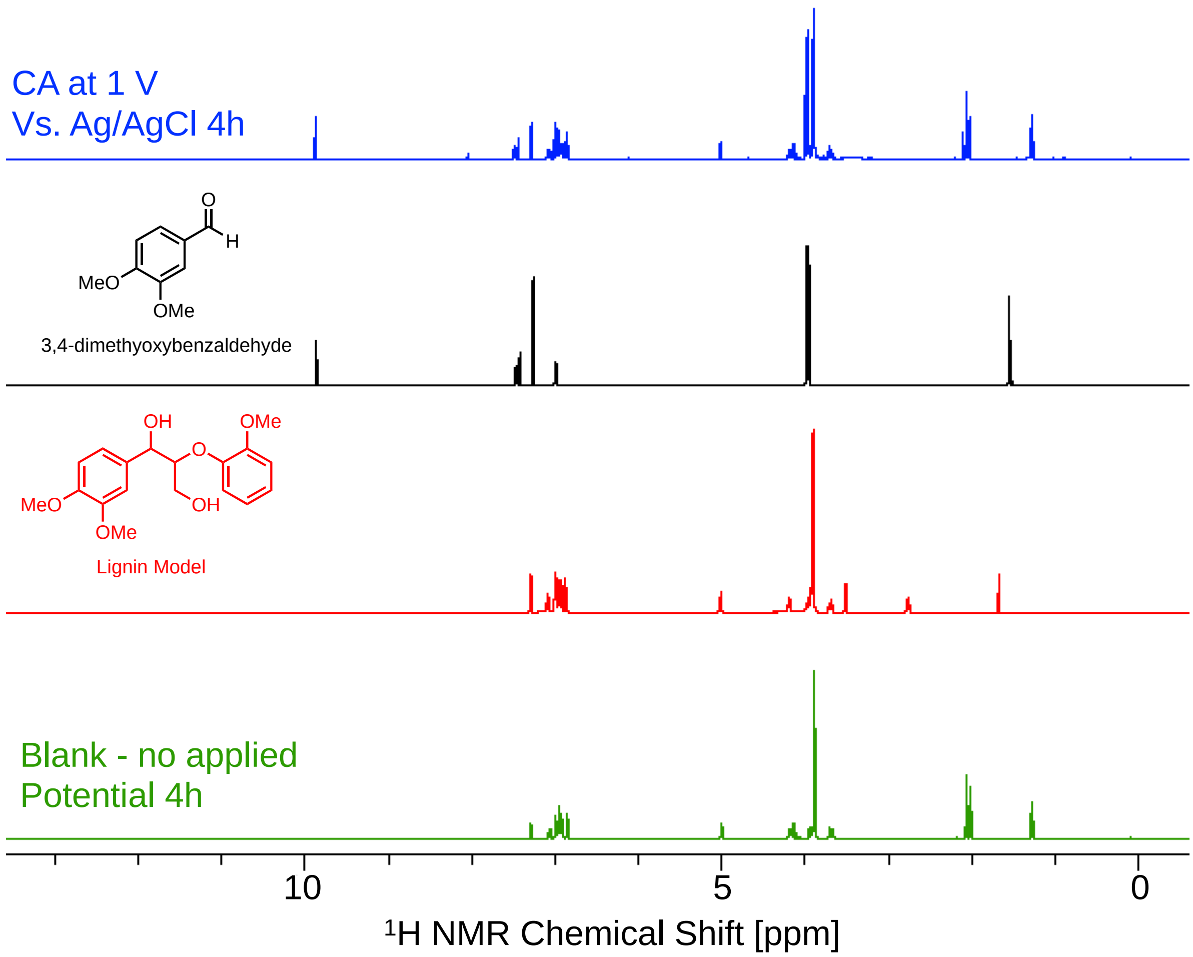

Figure S14. ^{1}H NMR of electrooxidised lignin model substrate in three-electrode setup. ^{1}H NMR of lignin model substrate after 4 h of electrolysis; ^{1}H NMR recorded in $CDCl_3$ and control experiment without any applied potential, and lignin model substrate and 3,4- dimethoxybenzaldehyde as reference. See Figure S17 for peak assignment. Reaction conditions: Chronoamperometry: 0.1 M Na_2CO_3, 0.01 M lignin model substrate in 1:1 MeCN:H_2O; +1 V vs. Ag/AgCl; 4 h WE: CP|MWCNT, CE: Pt foil, RE: Ag/AgCl, room temperature.

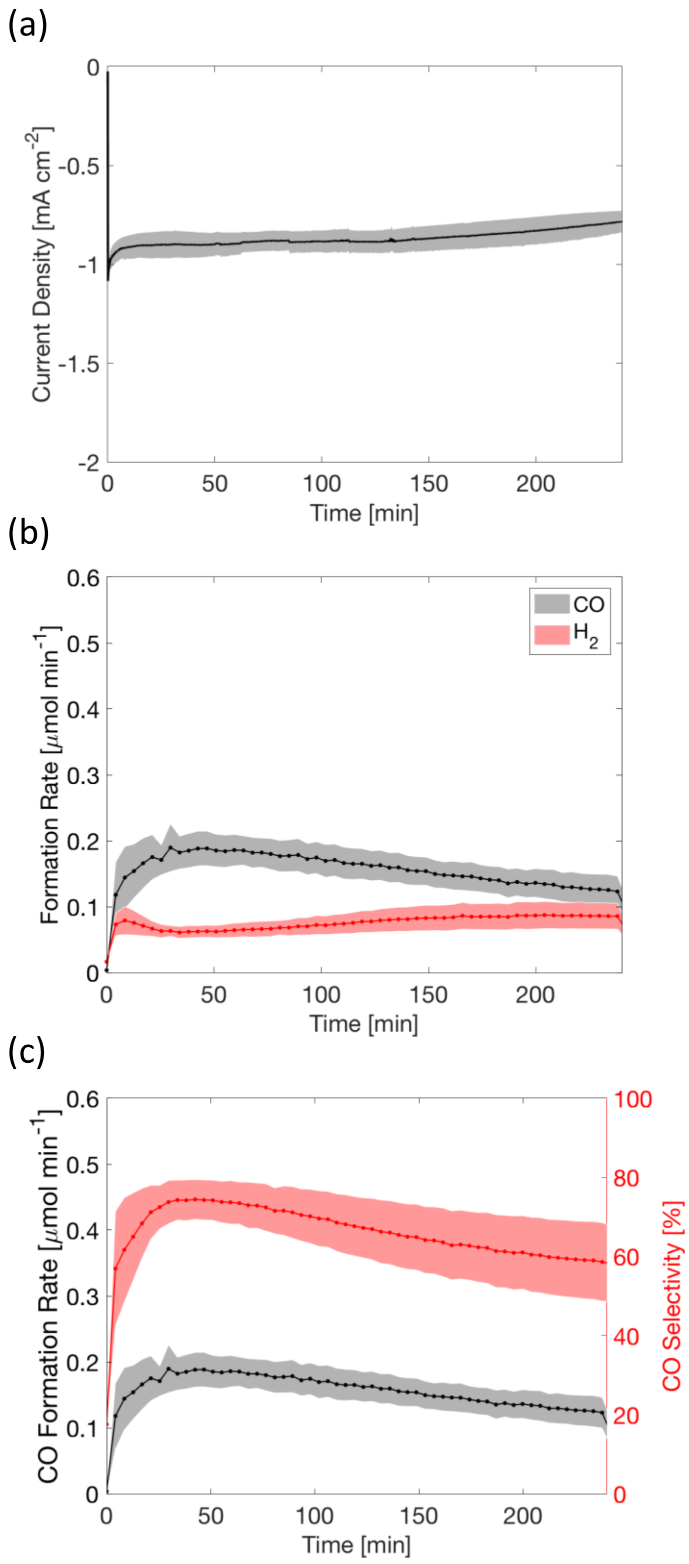

Figure S15. Electrolytic lignin model substrate oxidation coupled with low concentration CO_2 reduction. CO_2 electroreduction under 20 vol% CO_2 gas flow with CP|**CoP$_L$** coupled to lignin model substrate oxidation at the anode with CP|MWCNT. (a) Current density with time. (b) CO and H_2 formation rate obtained from online GC. (c) CO formation rate and selectivity. The shaded area represents the standard deviation. Reaction conditions: Electrolysis at U_{app} = –3 V. Anolyte: 0.1 M Na_2CO_3, 0.01 M lignin model substrate in 1:1 MeCN:H_2O; Anode: CP|MWCNT. Catholyte: 0.1 M $NaHCO_3$ in H_2O Flow of 20 vol% CO_2 (balanced with N_2) 9 mL min^{-1}; Cathode: CP|**CoP$_L$**, room temperature**.**

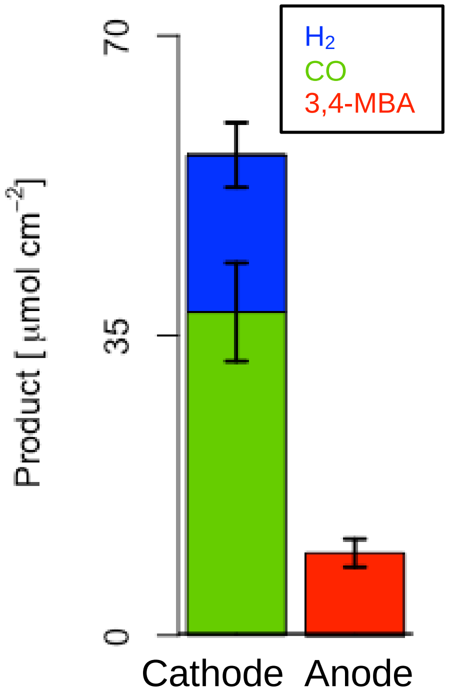

Figure S16. Products from electrochemical lignin model substrate oxidation coupled with low concentration CO_2 reduction. Products formed after 4 h of electrolysis. Reaction conditions: Electrolysis at U_{app} = –3 V. Anolyte: 0.1 M Na_2CO_3, 0.01 M Lignin Model in 1:1 MeCN:H_2O; Anode: CP|MWCNT. Catholyte: 0.1 M $NaHCO_3$ in H_2O Flow of 20 vol% CO_2 (balanced with N_2) 9 mL min^{-1}; Cathode: CP|**CoP**$_L$, room temperature.

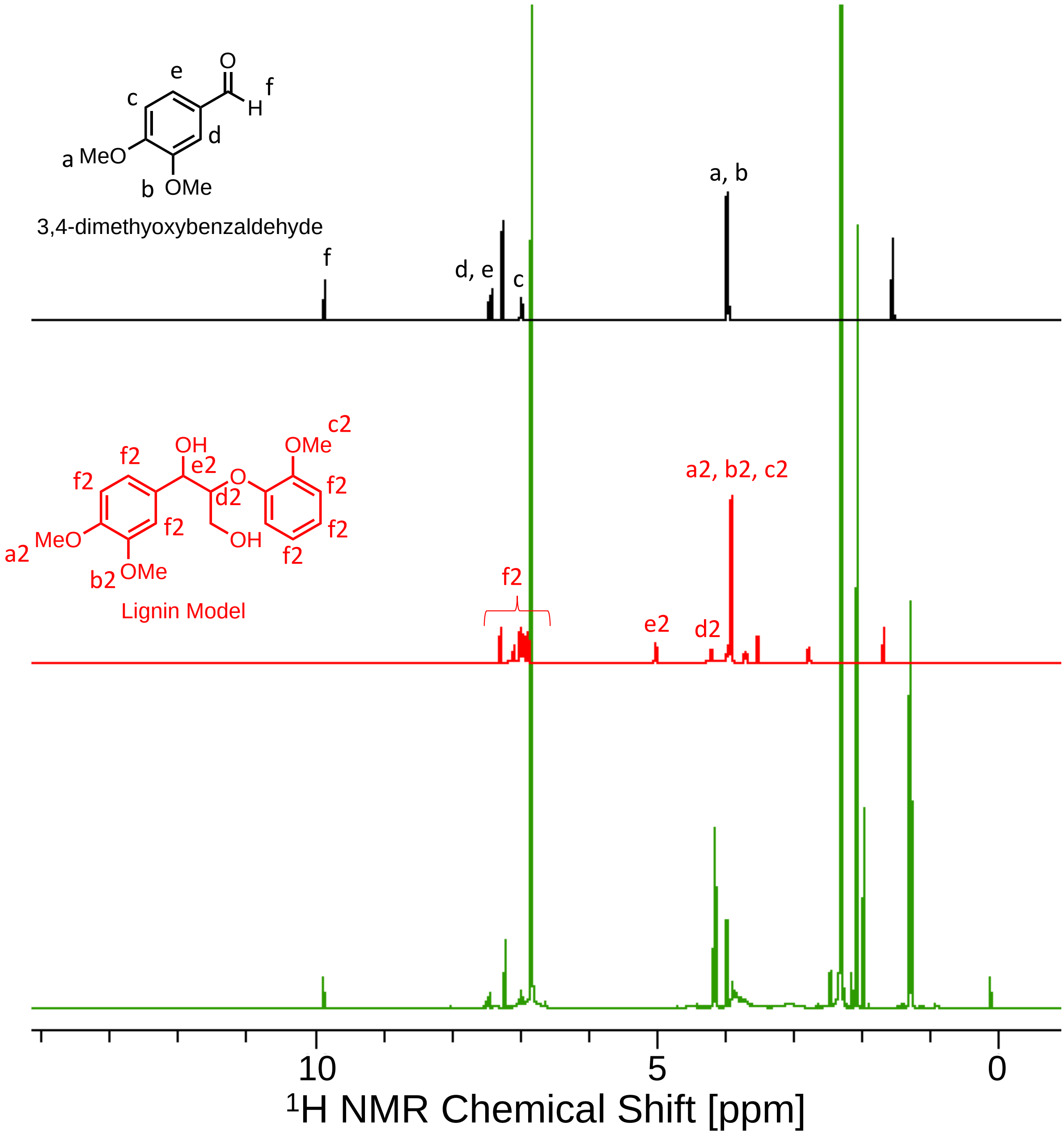

Figure S17. ^{1}H NMR of electrooxidised lignin model substrate in two-electrode setup. ^{1}H NMR of lignin model substrate after 4 h of electrolysis; ^{1}H NMR recorded in $CDCl_3$ with mesitylene as internal standard and 3,4-dimethoxybenzaldehyde and the lignin model substrate as reference spectra. Reaction conditions: Electrolysis at U_{app} = –3 V. Anolyte: 0.1 M Na_2CO_3, 0.01 M Lignin Model in 1:1 MeCN:H_2O; Anode: CP|MWCNT. Catholyte: 0.1 M $NaHCO_3$ in H_2O Flow of 20 vol% CO_2 (balanced with N_2) 9 mL min^{-1}; Cathode: CP|**CoP$_L$**, room temperature.

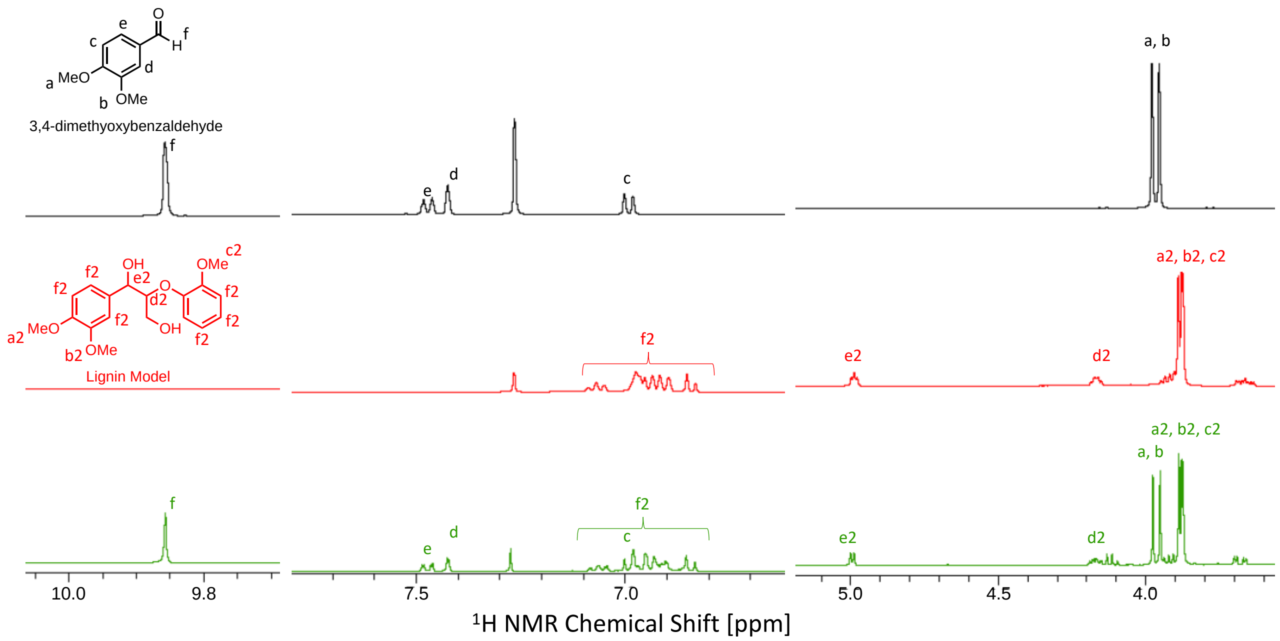

Figure S18. ^{1}H NMR of electrooxidised lignin model substrate in two-electrode setup. Zoomed in region ^{1}H NMR of lignin model substrate after 4 h of chronoamperometry; ^{1}H NMR recorded in $CDCl_3$ with 3,4-dimethoxybenzaldehyde and the lignin model substrate as reference. Reaction conditions: Chronoamperometry: 0.1 M Na_2CO_3, 0.01 M lignin model substrate in 1:1 MeCN:H_2O; +1 V vs. Ag/AgCl; 4 h; WE: CP|MWCNT, CE: Pt foil, RE: Ag/AgCl, room temperature.

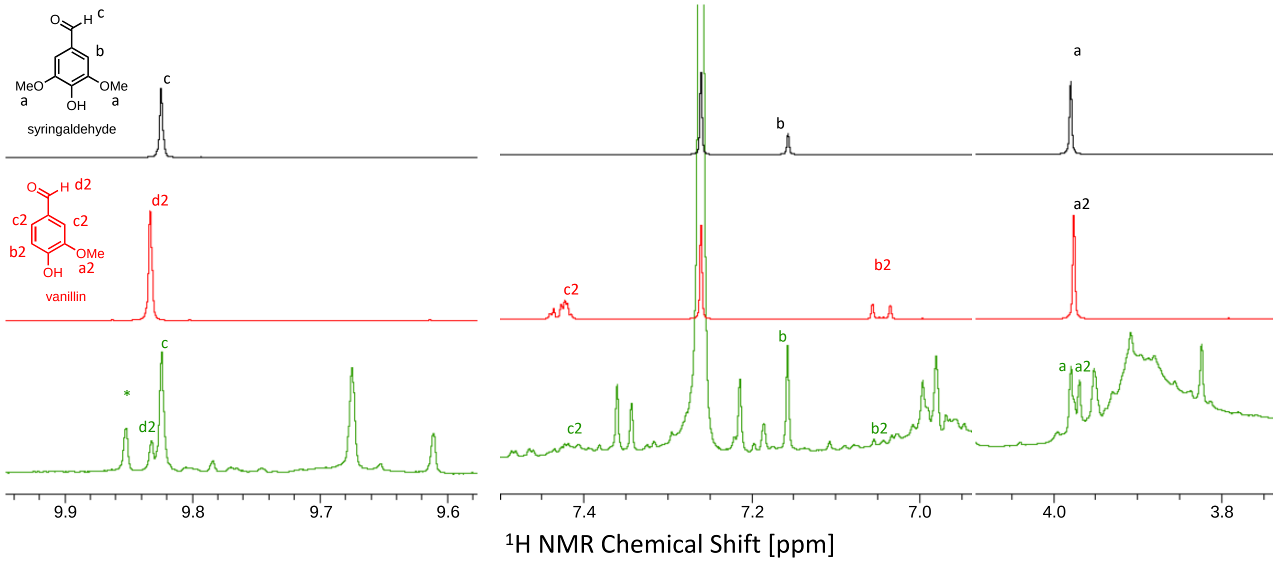

Figure S19. ^{1}H NMR of electrooxidised lignin in three-electrode setup. ^{1}H NMR of lignin after 4 h of chronoamperometry; ^{1}H NMR recorded in $CDCl_3$ with mesitylene as internal standard and vanillin and syringaldehyde as reference. * corresponds to additional aromatic aldehydes. Reaction conditions: Chronoamperometry: 0.1 M Na_2CO_3, lignin fraction obtained from pre-treating 250 mg Lignocellulose in 1:1 MeCN:H_2O; +1 V vs. Ag/AgCl; 4 h; WE: CP|MWCNT, CE: Pt foil, RE: Ag/AgCl, room temperature.

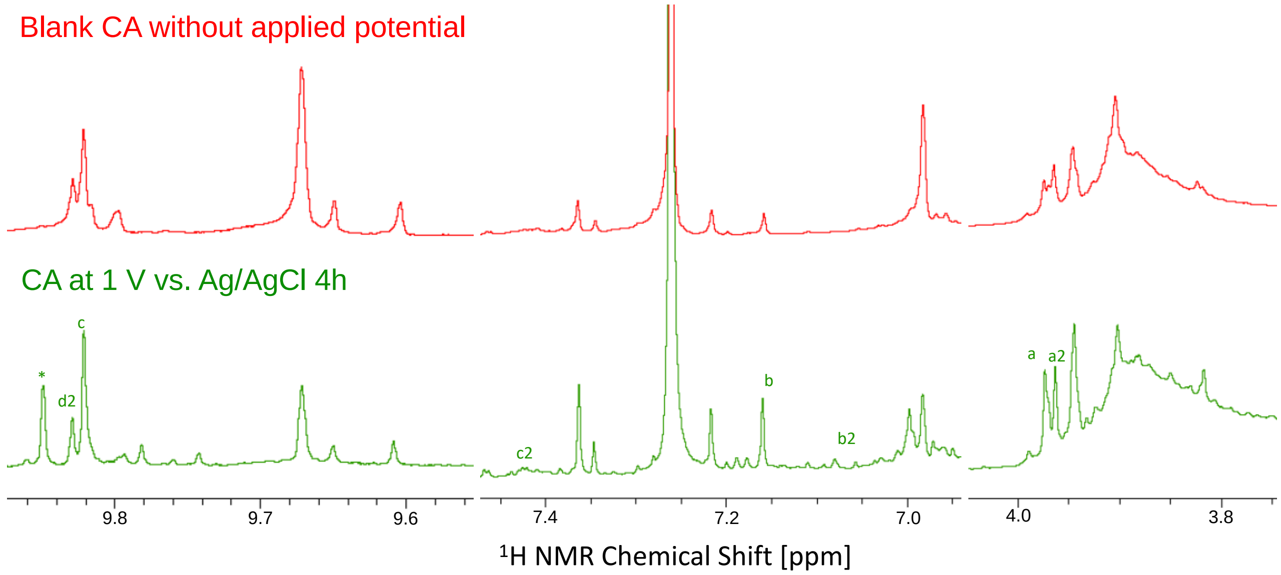

Figure S20. ^{1}H NMR of electrooxidised lignin in three-electrode setup. ^{1}H NMR of lignin after 4 h of electrolysis; ^{1}H NMR recorded in $CDCl_3$ and control experiment without any applied potential. a, b c are signals corresponding to syringaldehyde, a2, b2, c2 and d2 are signals corresponding to vanillin and * corresponds to additional aromatic aldehydes. Reaction conditions: Chronoamperometry: 0.1 M Na_2CO_3, lignin fraction obtained from pre-treating 250 mg Lignocellulose in 1:1 MeCN:H_2O; +1 V vs. Ag/AgCl; 4 h; WE: CP|MWCNT, CE: Pt foil, RE: Ag/AgCl.

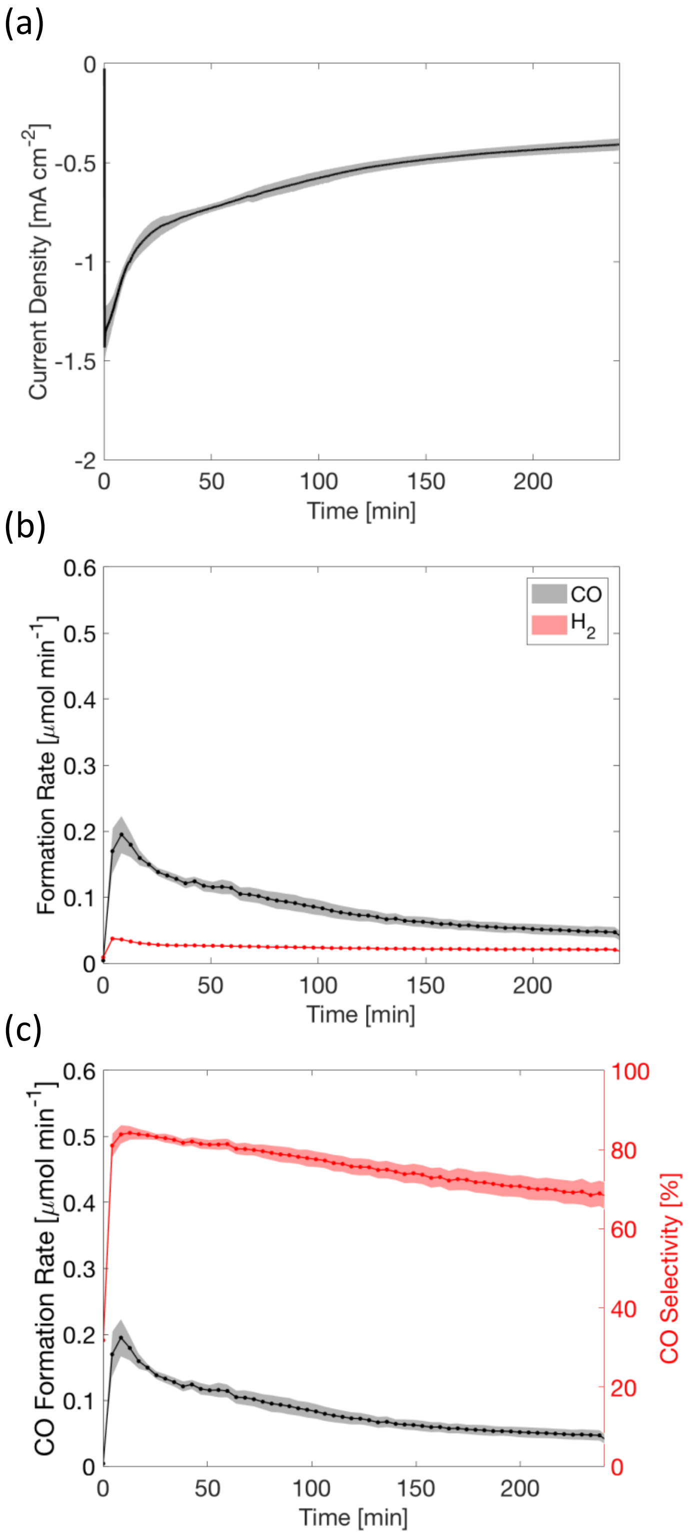

Figure S21. Electrochemical lignin oxidation coupled with low concentration CO_2 reduction. CO_2 electroreduction under 20 vol% CO_2 gas flow with CP|**CoP$_L$** coupled to lignin conversion at the anode with CP|MWCNT. (a) Current density with time. (b) CO and H_2 formation rate. (c) CO formation rate and selectivity. The shaded area represents the standard deviation. Reaction conditions: Electrolysis at U_{app} = –3 V. Anolyte: 0.1 M Na_2CO_3, lignin fraction obtained from pre-treating 250 mg Lignocellulose in 1:1 MeCN:H_2O; Anode: CP|MWCNT. Catholyte: 0.1 M $NaHCO_3$ in H_2O Flow of 20 vol% CO_2 (balanced with N_2) 9 mL min^{-1}; Cathode: CP|**CoP$_L$**.

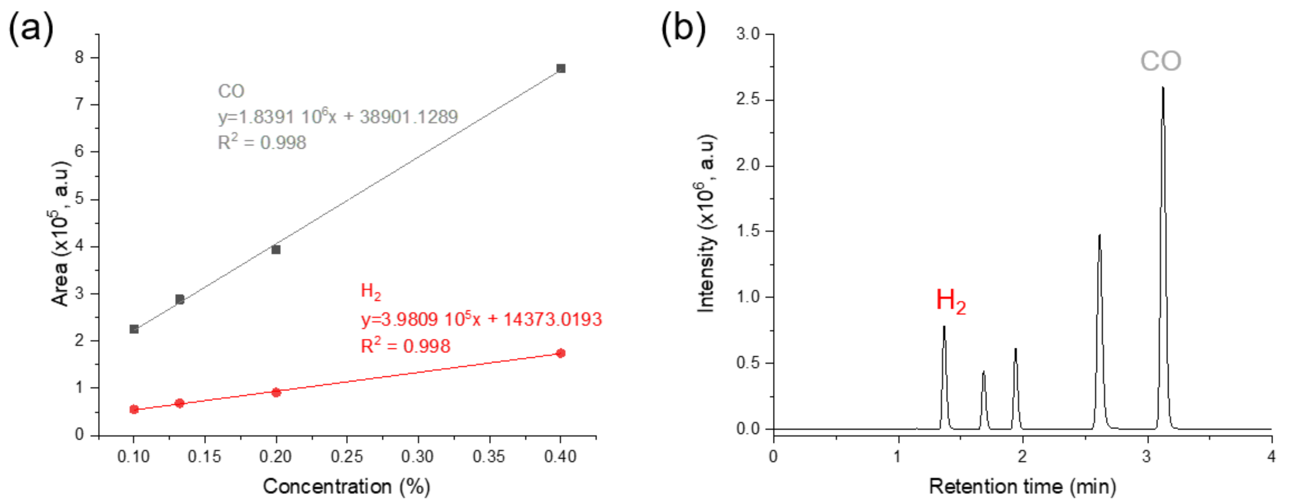

Figure S22. **H_2 and CO quantification.** (a) Calibration curve for H_2 (red) and CO (grey) quantification, and (b) spectra of H_2 (red) and CO (grey) at different concentrations obtained using gas chromatography.

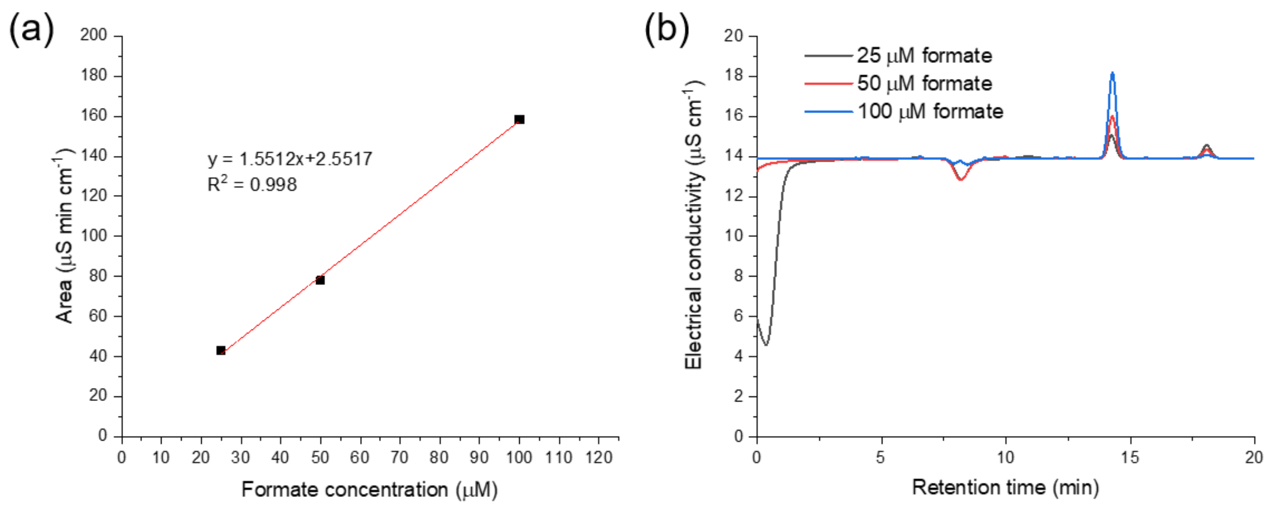

Figure S23. **Formate quantification.** (a) Calibration curve for formate quantification, and (b) spectra of formate at different concentrations obtained from ion chromatography (retention time 14.2 min).

Supporting Reference

1. Sahm, C. D.; Ucoski, G. M.; Roy, S.; Reisner, E., Automated and Continuous-Flow Platform to Analyze Semiconductor–Metal Complex Hybrid Systems for Photocatalytic CO_2 Reduction. *ACS Catal.* **2021,** *11*, 11266-11277.

End of Supporting Information
